# Quadruplex-Forming Motif Inserted into 3′UTR of *Ty1his3-AI* Retrotransposon Inhibits Retrotransposition in Yeast

**DOI:** 10.3390/biology10040347

**Published:** 2021-04-20

**Authors:** Viktor Tokan, Jose Luis Rodriguez Lorenzo, Pavel Jedlicka, Iva Kejnovska, Roman Hobza, Eduard Kejnovsky

**Affiliations:** 1 Department of Plant Developmental Genetics, Institute of Biophysics of the Czech Academy of Sciences, Kralovopolska 135, 61200 Brno, Czech Republic; viktor.tokan@ibp.cz (V.T.); rodriguez@ibp.cz (J.L.R.L.); jedlicka@ibp.cz (P.J.); hobza@ibp.cz (R.H.); 2 Department of Biophysics of Nucleic Acids, Institute of Biophysics of the Czech Academy of Sciences, Kralovopolska 135, 61200 Brno, Czech Republic; kejnovska@ibp.cz

**Keywords:** *Ty1* LTR retrotransposon, G-quadruplex, retrotransposition, N-methyl mesoporphyrin (NMM), Pif1 helicase, yeast

## Abstract

**Simple Summary:**

Four-stranded DNA (guanine quadruplex) can regulate many cellular processes such as replication, recombination and also gene expression. This study shows that four-stranded DNA can also affect the activity of mobile genetic elements (transposable elements). We inserted a quadruplex-forming motif into a non-coding region of the Ty1 transposable element and tested its activity in yeast cells. We found that the quadruplex inhibited Ty1 jumping. Especially when the quadruplex was formed on the transcribed strand, the amplification of the element was hindered. Our study shows that DNA conformation can tune the activity of mobile genetic elements that in turn shape eukaryotic genomes during evolution.

**Abstract:**

Guanine quadruplexes (G4s) serve as regulators of replication, recombination and gene expression. G4 motifs have been recently identified in LTR retrotransposons, but their role in the retrotransposon life-cycle is yet to be understood. Therefore, we inserted G4s into the 3′UTR of *Ty1his3-AI* retrotransposon and measured the frequency of retrotransposition in yeast strains BY4741, Y00509 (without Pif1 helicase) and with G4-stabilization by N-methyl mesoporphyrin IX (NMM) treatment. We evaluated the impact of G4s on mRNA levels by RT-qPCR and products of reverse transcription by Southern blot analysis. We found that the presence of G4 inhibited *Ty1his3-AI* retrotransposition. The effect was stronger when G4s were on a transcription template strand which leads to reverse transcription interruption. Both NMM and Pif1p deficiency reduced the retrotransposition irrespective of the presence of a G4 motif in the *Ty1his3-AI* element. Quantity of mRNA and products of reverse transcription did not fully explain the impact of G4s on *Ty1his3-AI* retrotransposition indicating that G4s probably affect some other steps of the retrotransposon life-cycle (e.g., translation, VLP formation, integration). Our results suggest that G4 DNA conformation can tune the activity of mobile genetic elements that in turn contribute to shaping the eukaryotic genomes.

## 1. Introduction

Guanine-rich sequences (with consensus motive $G_{\geq 3}N_{1-7}G_{\geq 3}N_{1-7}G_{\geq 3}N_{1-7}G_{\geq 3}$) are capable of forming four-stranded DNA and RNA structures called G-quadruplexes (G4s, recently reviewed in [1]). Since their discovery by Henderson in the 1980s [2], G4s rapidly gained attention among scientists. The first structural studies were focused mostly on quadruplex molecularity and topology, but later in vitro and In Vivo studies showed the biological importance of quadruplexes in a myriad of cellular processes serving as regulators of replication, telomere maintenance, transcription, translation, recombination and chromatin organization [3,4,5].

Quadruplex-forming sequences (QSs) are abundant in eukaryotic genomes, e.g., almost 150 thousand putative non-telomeric QSs were found in the maize genome [6]. In the human genome, more than 700 thousand distinct G4 structures were identified by the G4-Seq approach [7] while only about 25 thousand were found by Single-Molecule Real-Time (SMRT) technology [8].

QSs are often present in regulatory regions of genes but we have recently found accumulation of QSs within human and plant transposable elements (TEs; [9,10,11]). Transposable elements constitute a significant proportion (up to 80%) of most eukaryotic genomes [12,13,14,15]. We have shown that potential quadruplex-forming motifs are present in regulatory regions of transposable elements and are more abundant in evolutionarily young elements [9,10]. However, the role of quadruplexes in the retrotransposon life-cycle is far from understood.

*Ty1* elements, LTR retrotransposons of the *Ty1*/copia superfamily, belong to the most studied transposable elements in yeast. They represent a useful model system for studies of retrotransposition regulation [16]. The engineered *Ty1* element containing the *HIS3* gene with an artificial intron (AI), that is spliced before reverse transcription, enables the detection of new *Ty1* retrotransposition events in His3^+^ colonies [17]. Such a system referred to as *Ty1his3-AI* has the potential for inserting additional sequences (e.g., QSs) into *Ty1* and studying their effect on retrotransposition.

Previously, we have shown the effect of LTR located G4s on a downstream reporter gene [18]. Here, we inserted a quadruplex-forming motif into the 3′UTR region of the *Ty1his3-AI* retrotransposon and studied its effect on retrotransposition in yeast as well as the impact of the G4-stabilizing drug N-methyl mesoporphyrin (NMM) and an absence of Pif1 helicase, an enzyme which unwinds quadruplexes [19,20,21]. We also evaluated how individual steps of *Ty1his3-AI* retrotransposition are affected by G4 formation.

## 2. Materials and Methods

### 2.1. Plasmid Construction

All plasmids were based on the pG*Ty1his3-AI* plasmid (Addgene plasmid # 62228; http://n2t.net/addgene:62228 (accessed on 5 January 2018); RRID:Addgene_62228). In order to clone the sequences of interest into the plasmid, a *SacII* recognition site was introduced into the *Ty1his3-AI* 3′UTR using a single mutagenic primer GAGGATCCCGCGGGGAGCTC and the QuikChange Multi Site-Directed Mutagenesis Kit (Agilent, Santa Clara, CA, USA). The mutant product was electroporated into XL-1 blue competent cells (Agilent) and the mutation was verified by *SacII* digestion and sequencing. The resulting plasmid pV*Ty1* was then digested by SacII, blunted, dephosphorylated and used for cloning annealed oligonucleotides (Table S1). To anneal the oligonucleotides, 300 pmol of each sense and antisense strands were treated for 90 min at 37 °C with 20 units of T4 polynucleotide kinase (New England Biolabs, Ipswich, MA, USA). After incubation, the reactions were placed in a boiling water bath for 2 min and allowed to cool to room temperature slowly. Then, 1 µL of 100× diluted annealed oligonucleotides together with 50 ng of plasmid was used for ligation. Copy-number and orientation of cloned sequences were verified by sequencing.

### 2.2. Circular Dichroism Spectroscopy, PAGE

Circular dichroism and polyacrylamide gel electrophoresis (PAGE) were performed as described in [9]. The CD measurements were carried out at 22 °C in accordance with yeast growth conditions. NMM was added to DNA and potassium solution in increments to reach the final concentration of 0.25, 0.625, 1.25, 1.875, 2.5 and 7.5 µM representing 0.1, 0.25, 0.5, 0.75, 1 and 3 molar ratios to oligonucleotides, respectively.

### 2.3. UV Absorption Spectroscopy and Thermal Melting

UV absorption spectra were collected on a UV/VIS spectrometer Varian Cary 4000 (Mulgrave, Oakleigh, Australia) in the range of 230–330 nm in 1 cm cells. The temperature was increased/decreased in 1 °C steps and the samples were equilibrated for 2 min before each measurement. The melting curves were expressed as the change in molar absorption ϵ [M${}^{-1}$cm${}^{-1}$] at 297 nm with temperature and the Tm values were calculated as half of the nature/denature state from the normalized melting dependencies.

### 2.4. Retromobility Assay

**Figure 1:**
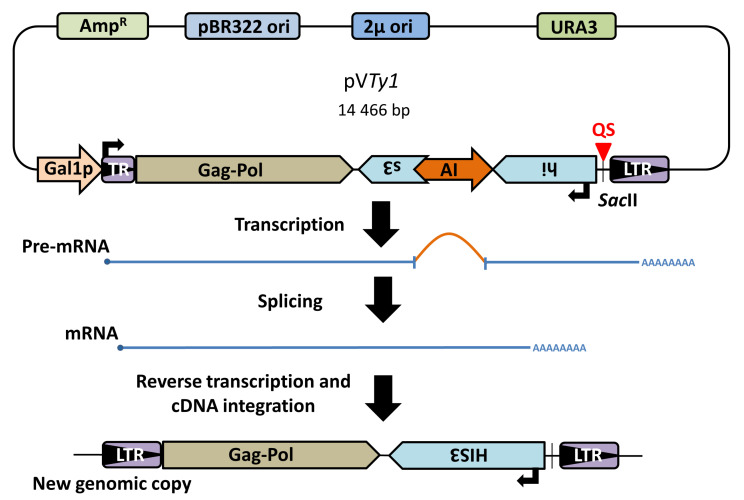
Schema of plasmid containing *Ty1his3AI* retrotransposon and element retrotransposition. Plasmid borne chimeric *Ty1* element contains retrotransposition indicating *HIS3* gene in opposite orientation with respect to *Ty1* transcription. The *HIS3* gene is disrupted by an artificial intron (AI), which is spliceable only when transcription starts from an inducible Gal1 promoter (Gal1p). After splicing and reverse transcription, the new *Ty1* genomic copy contains functional *HIS3* and yeast can be grown on selective plates without histidine. TR-5′ truncated LTR.

Yeast strains BY4741 and Y00509 (BY4741; *MATa*; *ura3*Δ*0*; *leu2*Δ*0*; *his3*Δ*1*; *met15*Δ*0*; *pif1::kanMX4*; EUROSCARF) were transformed by pV*Ty1*-based plasmids (Figure 1). Three to four single colonies of transformants were inoculated into 1500 µL SC-URA, 2% glucose and shaken at 250 rpm at 30 °C over-night. Next day, 100 µL of the culture was used for inoculation of 1500 µL SC-URA, 0.1% glucose, 2% raffinose and grown as previously. In the case of NMM (N-methylmesoporphyrin IX, Frontier Scientific) treatment, it was added to the final 8 µM concentration, to ensure G4 stabilization prior to *Ty1* retrotransposition induction. The following day, 100 µL of 1000× diluted over-night culture was used to inoculate 1500 µL SC-URA, 2% raffinose, 2% galactose, (8 µM NMM) and shaken at 250 rpm at 22 °C for several days until saturation. The 8 µM NMM concentration was chosen based on literature [22]. Then 100 µL of each culture was plated in serial dilutions on SC-HIS and YPD plates for colony counting. Best dilutions were 500× for SC-HIS and 100,000× for YPD, 2–3 plates were counted for each colony and dilution. The retrotransposition frequency was calculated as His positive CFU portion of all CFUs. One-way ANOVA (for absolute retrotransposition frequencies) followed by Tukey’s HSD test (*p* ≤ 0.05) and Two-way ANOVA (for relative retrotransposition frequencies) followed by Dunnett’s multiple comparisons test (*p* ≤ 0.05) were performed using GraphPad Prism version 8.0 for Windows (GraphPad Software, La Jolla, CA, USA, www.graphpad.com (accessed on 2 June 2019)) at a 0.05 significance level.

### 2.5. RT-qPCR

For RNA isolation, 10 transformants were grown in the same way as for the transposition assay. RNA isolation was then performed in a pool containing 1 mL of each culture. Hot acidic phenol extraction protocol was used for RNA isolation [23], but with the RNA being precipitated by LiCl and repurified using RNAzol^®^ RT (Molecular Research Center, Inc.Cincinnati, OH, USA) with 4-bromoanisole (Sigma Aldrich, St. Louis, MO, USA). This protocol produced RNA with only a very small amount of DNA contamination that was then completely removed by TURBO DNA-free™ Kit (Invitrogen, Carlsbad, CA, USA). Reverse transcription was done using High-Capacity RNA-to-cDNA™ Kit (Applied Biosystems, Foster City, CA, USA). RT-qPCR was performed in triplicate with Rotor-Gene Q (Qiagen, Hilden, Germany) and SensiFAST™ 2X HRM kit (Bioline, London, UK), 10 ng of cDNA and 5 µM of each primer (Table S1). Data analysis and processing was done in LinReg software (Heart Failure Research Center, Amsterdam, NL; [24]). Statistics were performed in R Studio, V.1.2.1335 (R Development Core Team; Boston, MA, USA; [25]), using the powerTransform function for transformation followed by Univariate analysis of variance (ANOVA) with GLM procedure to decipher factor interactions and post hoc Tukey’s HSD test (*p* ≤ 0.05) to carry out multiple comparisons.

### 2.6. cDNA Analysis

Yeast cultures were prepared the same as for the retromobility assay except for an increase in volume to 5 ml SC-URA (2% rafinose, 2% galactose). Two controls were included: cells with pV*Ty1* without galactose induction (glucose only) and cells without plasmid but with galactose induction (SC with all amino acids). Spheroplasts were prepared by incubation of cells with 1 mg/mL of Zymolyase 20T (MP Biomedicals) at 37 °C for 60 min in 0.9 sorbitol, 0.1 M EDTA containing 1 µL of ß-mercaptoethanol per mL (Sigma Aldrich) and 0.1 mg/mL RNase A (Qiagen). DNA from spheroplasts was extracted by incubation with CTAB extraction buffer for 45 min at 62 °C (2% CTAB, 2M NaCl, 10mM EDTA, 50 mM HEPES) followed by two chloroform extractions and isopropanol precipitation. About 3 µg of total DNA were digested by *AflII* and subjected to electrophoresis on 1% agarose and transferred to Hybond-N^+^ (Amersham, UK). The membrane was hybridized with *HIS3* PCR product (Table S1) labeled by $\alpha^{32}$P (dATP) using Prime-It II Random Primer Labeling Kit (Agilent). The ${}^{32}$P activity was analyzed by Typhoon FLA9000 and ImageQuant TL v8.1.0.0 software (GE Healthcare; Chicago, IL, USA).

## 3. Results

### 3.1. Characterization and Biophysical Properties of G4 Sequences

In order to test the effect of G4 on retrotransposition we choose a modified chimeric *Ty1his3-AI* retrotransposon system ([17]; see Materials and Methods for details). As a first step, we analyzed the *Ty1his3-AI* element (File S1) for its natural G4 forming ability using PQS finder [26]. The default setting, copying human G4-seq detected quadruplexes (min. score 47), did not propose any potential quadruplex sequence (PQS). To confirm this absence we applied a suboptimal min. score of 25 which resulted in the identification of 4 PQSs all on the template strand (Figure S1). However, none of the sequences contained an undisrupted track of 3 guanines, a typical prerequisite for G4 formation. According to both CD spectroscopy and thermal UV melting assays, none of these sequences form orderly structures in vitro, as suggested by the low amplitude of ellipticity (thermal melting data not shown).

**Figure 2:**
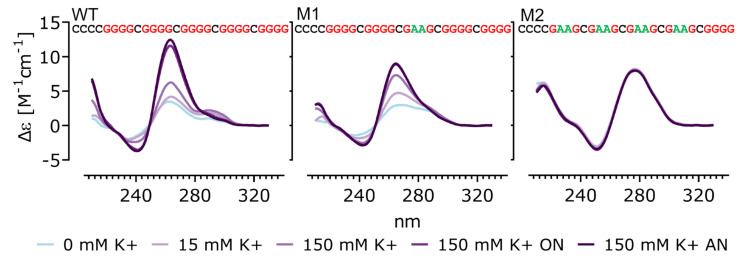
CD spectra of inserted sequences into *Ty1his3-AI*. CD spectra obtained in an increasing potassium concentration showing formation of quadruplex in WT and M1 but not in M2 (ON—overnight, AN—annealed). The sequences of tested oligonucleotides are shown with G-tracts highlighted by red and G to A substitutions by green color.

Following the confirmation that quadruplexes were not formed by motifs present within *Ty1his3-AI*, we proceeded with inserting quadruplex-forming sequences (QSs) as well as control sequences into the element. The QS originated from the LTR retrotransposon Machiavelli family found in *Zea mays* B73 [27], and the control sequences were derived from the QS by replacing a number of guanines with adenines (Figure 2).

In order to characterize the effect of guanine substitutions on G4 formation, stability and interaction with the G4 stabilizing drug NMM, circular dichroism spectroscopy was used with a combination of UV absorption spectroscopy, thermal melting assay and polyacrylamide gel electrophoresis (PAGE). All tested oligonucleotides represented the G-rich strand and also contained an additional G-track present on the plasmid carrying *Ty1his3-AI* element (Figure 2). Both the WT and M1 sequences formed parallel-stranded G4s suggested by the development of a typical CD spectra in increasing potassium concentration with peaks at 260 and 240 nm [28]. The CD spectra of the M2 sequence were not affected by increasing potassium concentration and were shifted to longer wavelengths, suggesting B-DNA formation. Native PAGE revealed that WT, as well as M1, formed monomolecular G-quadruplexes where WT also contained a bimolecular fraction after annealing in 150 mM potassium (Figure S2). M2 produced a clear strong band on native PAGE gel, confirming B form hairpin formation.

**Figure 3:**
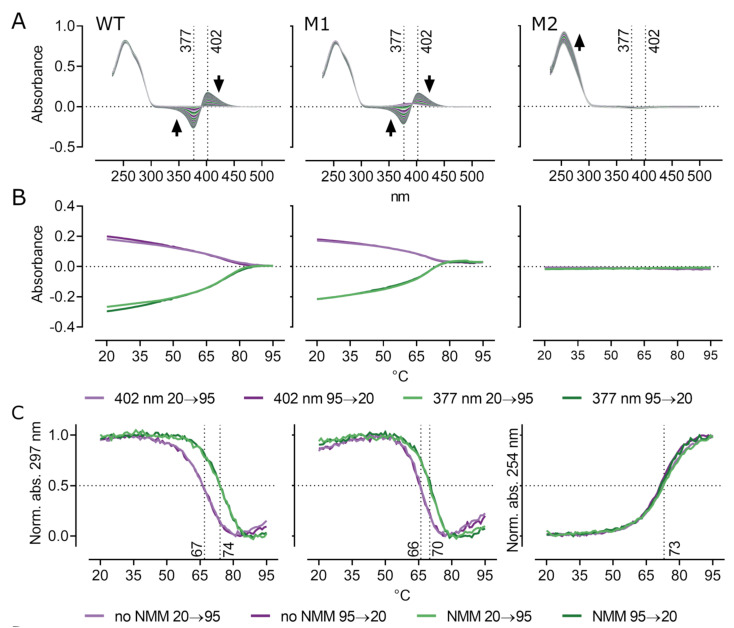
UV absorption spectroscopy and thermal melting. (**A**) Full absorption spectra obtained during heating from 20 to 95 °C in 15 mM potassium and 7.5 µM NMM show typical peaks at 377 and 402 nm of free and G4-bound NMM, respectively. Black arrows indicate peak development with increasing temperature. (**B**) Absorbance at 377 and 402 nm plotted against temperature revealing a disruption of NMM-G4 interaction due to the increase in temperature. (**C**) Comparison of normalized absorbance of WT and M1 at 297 nm shows the effect of NMM on G4 melting temperature and G4 stabilization by NMM in 15 mM potassium. Melting curves for M2 are calculated as normalized absorbance at 254 nm.

To test the effect of NMM on G4 formation and stability, we first added NMM increments to oligonucleotides (up to 3:1 molar ratio) supplemented with only 15 mM K^+^. The CD spectra showed that NMM sped up the G4 formation however the effect on the final structures was negligible (Figure S3). UV absorption spectroscopy and thermal melting assay showed that addition of NMM to WT and M1 gave rise to a typical absorption peak at 402 nm, indicating an interaction of NMM with G4s (Figure 3A; [21]). This absorption peak reached zero values with an increasing temperature as the G4 melted and the interaction was disrupted (Figure 3B). Absorption maxima of free NMM at 377 nm behaved in the opposite manner with respect to temperature. Note that due to NMM being also in a blank sample, the free NMM signal reached negative values when there was G4–NMM interaction in the sample. The spectrum of M2 was not changed by NMM. The melting temperatures (Tm) of G4 forming oligonucleotides were calculated at 297 nm and revealed that NMM increased Tm by 7 °C in WT and by 4 °C in M1 suggesting that NMM stabilized tested quadruplexes (Figure 3C). The melting temperature of M2, which does not form G4, was calculated at 254 nm and irrespective of NMM addition Tm remained at 73 °C. A different wavelength for melting temperature calculation of M2 was used because of negligible differences of A_297_ with respect to temperature changes.

In order to meet the physiological potassium concentration, we also tested the effect of NMM in 150 mM K^+^ which produced similar results as in lower potassium concentration (Figure S4). As a result of the high G4 stability in physiological potassium concentration, it was not possible to precisely determine melting temperatures of the oligonucleotides and therefore these are only roughly estimated. Both G4 forming WT and M1 oligonucleotides adopted a stable G4. The melting temperature of the M1 oligonucleotide was 83 °C and the hairpin formed by M2 was also 83 °C, whereas the WT did not completely melt even at 99 °C. Nevertheless, we still observed typical NMM related absorption peaks and shift of the melting curves towards higher temperatures suggesting G4 stabilization by NMM also in 150 mM potassium. Interestingly, while WT was more stabilized by NMM than M1 in 15 mM potassium, the shift of melting curves in 150 mM potassium was more prominent in M1.

### 3.2. Inhibition of Retrotransposition—Effect of Strand Orientation, Pif1 Deficiency and NMM

**Figure 4:**
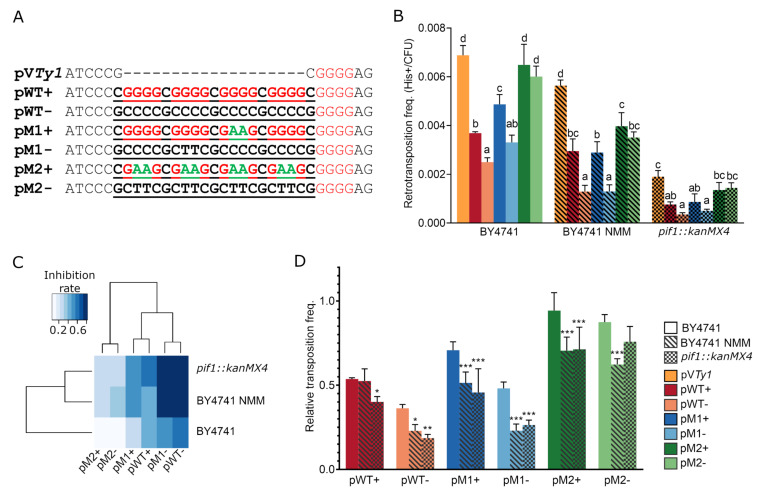
Frequency of *Ty1his3-AI* retrotransposition. (**A**) Sequences cloned (underlined) into *SacII* recognition site located in 3′UTR of *Ty1his3-AI*. Guanine blocks are highlighted by red and G to A substitutions by green color. (**B**) Absolute retrotransposition frequency of *Ty1his3-AI* (pV*Ty1*) containing G-quadruplexes (pWT and pM1) as well as control sequences (pM2), demonstrate the inhibitory effect of G4s as well as Pif1p deficiency and NMM treatment. Columns represent mean ± SD (*n* = 3–4 colonies) of retrotransposition frequency. Significantly different transposition frequencies within each treatment/yeast strain are indicated by different letters. (One way ANOVA, Tukey’s HSD test; *p*≤ 0.05). (**C**) Heatmap of inhibitory effect of inserted sequences combined with NMM treatment and Pif1p deficiency on retrotransposition. (**D**) Relative *Ty1his3-AI* retrotransposition frequency normalized to pV*Ty1* revealing an inhibitory effect of NMM treatment or Pif1 helicase deficiency (*pif1::kanMX4*). Statistical differences are with respect to BY4741 (One way ANOVA, Dunnett’s test). Columns represent mean ± SD (*n* = 3–4 colonies) of retrotransposition frequency. * *p*-value ≤ 0.05; ** *p*-value ≤ 0.01; *** *p*-value ≤ 0.001.

As mentioned above, we cloned the QS and control sequences into the modified *Ty1his3-AI*. This element is carried by pV*Ty1* plasmid and contains a *SacII* recognition site in the 3′UTR 42 bp upstream of the polypurine tract (Figure S1A). Tested sequences were cloned in both orientations. Plasmids carrying wild-type QSs were denoted as pWT+ and pWT− depending on the presence of the G rich sequence on either the coding or template strand, respectively (Figure 4A). Similarly the plasmids containing mutated G4-forming motifs were named pM1+/− and pM2+/−. Using the plasmid constructs containing G4-forming motifs (pWT+/− and pM1+/−), constructs where G to A substitutions prevented G4 formation (pM2+/−) and a control without inserted QS (pV*Ty1*), we measured the retrotransposition frequency in *Saccharomyces cerevisiae* strain BY4741 (Figure 4B). We observed that the presence of quadruplexes strongly inhibited retrotransposition, especially when the G rich strand was the template for transcription (constructs pWT− and pM1−). The presence of M2 sequence showed no significant reduction of retrotransposition compared to pV*Ty1* and also the orientation of M2 sequence was not important (Tukey’s HSD test, *p* ≤ 0.05).

In order to further enhance the effect of G4s, we treated the BY4741 cells with G4 stabilizing ligand NMM and and also used an isogenic strain Y00509 lacking the G4 unwinding helicase Pif1 (containing *pif1::kanMX4* mutation). The inhibitory effect of both G4 stabilizing conditions on *Ty1his3-AI* retrotransposition is scaled in the heatmap of aligned and clustered data normalized to pV*Ty1* control (Figure 4C). The most suppressed retrotransposition was recorded in BY4741 cells treated with NMM (BY4741 NMM) and in the strain lacking Pif1 helicase carrying constructs pWT− and pM1−. However, two-way analysis of variance revealed a higher significance of plasmid construct modification than the yeast treatments (*p* < 2 × 10^−16^ and *p* = 5.51 × 10^−8^, respectively) suggesting that the presence of G4 was critical and that G-quadruplex orientation was more important than its stability.

Both the NMM treatment and the absence of Pif1 helicase reduced the overall retrotransposition frequency, even in the case of pV*Ty1* (Figure 4B). In particular, the absence of Pif1 helicase showed much stronger effect than NMM treatment and reduced the transposition frequency by roughly 3.6-fold compared to untreated BY4741. Surprisingly, we also observed a significant inhibitory effect of both NMM treatment and *pif1::kanMX4* mutation on the pM2+ and pM2− relative retrotransposition frequency normalized to pV*Ty1* (Dunnett’s test, *p* ≤ 0.05; Figure 4D).

### 3.3. The Effect of G4 and Its Stabilization on mRNA Levels and Products of Reverse Transcription

**Figure 5:**
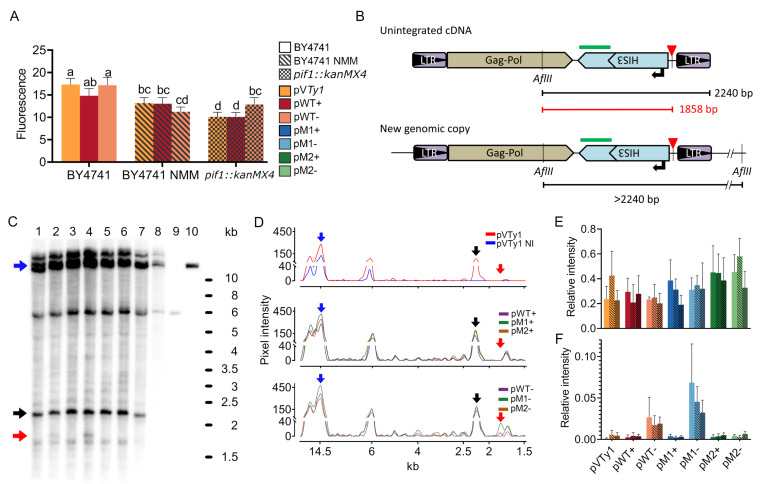
Measurement of *Ty1his3-AI* mRNA levels by real-time PCR and analysis of reverse transcription products by Southern blot. (**A**) mRNA relative quantities of plasmid borne *Ty1his3-AI* measured by real-time PCR showing only a slight inhibitory effect of G4 but also overall reduction of mRNA by both NMM treatment and *pif1::kanMX4*. The mRNA levels were normalized to the plasmid-borne URA3 mRNA. Significantly different expression levels are indicated by different letters (One way ANOVA, Tukey’s HSD test; *p* ≤ 0.05). (**B**) Schematic of Southern blot design of unintegrated cDNA and genomic copy indicating hybridization probe position (green rectangle) and relevant *AflII* sites. The probe detected a 2240 bp AflII fragment corresponding to unintegrated *Ty1his3-AI* cDNA and various longer fragments originating from the junction of *Ty1his3-AI* and chromosomal DNA. (**C**) Southern blot analysis of *AflII*-digested total cellular DNA of cultures induced for *Ty1his3-AI* retrotransposition by cultivation at 22 °C in galactose containing media (except for line 8, glucose). Plasmid, cDNA and interrupted products of reverse transcription are indicated by blue, black and red arrows, respectively. Band around 6 kb originates from a gDNA fragment containing *his3*Δ*1*. Lines: (1) pWT+; (2) pWT−; (3) pM1+; (4) pM1−; (5) pM2+; (6) pM2−; (7) pV*Ty1*; (8) pV*Ty1* non-induced (NI); (9) BY4741 without plasmid; (10) pV*Ty1* isolated plasmid. (**D**) Signal intensity of selected lines. For better visualization, the curves were aligned so that corresponding peaks match. (**E**) Quantification of unintegrated cDNAs normalized to plasmid intensity. (**F**) Quantification of interrupted products of reverse transcription normalized to plasmid intensity. Plotted values are means and standard deviations of three biological replicates (independent cultures), except for NMM representing only two replicates.

To assess which step of the *Ty1his3-AI* life-cycle was affected by G4 formation we first measured the mRNA quantity by real-time PCR for possible G4 effect on transcription and/or mRNA stability (Figure 5A). We tested a limited sample set consisting of 10 pooled induced cultures of control pV*Ty1* and pWT+/− with a combination of both NMM treatment and deficiency of Pif1 helicase. We observed an overall reduction of mRNA quantity by NMM treatment as well as by Pif1p deficiency. However, there was not any common effect of both the presence of G4 and G-rich strand orientation that we observed in the case of retrotransposition frequency. This shows that mRNA level is not affected by G4 but rather that both Pif1 helicase deficiency and NMM treatment reduce *Ty1his3-AI* mRNA levels.

Consequently, we used Southern blot analysis to assess the effect of G4 on products of reverse transcription (Figure 5B–F). We utilized a similar approach as [29] hybridizing *Ty1his3-AI* specific probe on *AflII* digested total genomic DNA. In this approach, an unintegrated extrachromosomal cDNA fragment is the shortest (2240 bp) since all other fragments originating from genomic DNA also contain a junction with surrounding chromosomal DNA and hence are longer (Figure 5B). We used a probe homologous to the coding part of the *HIS3* gene. Such probe design ensures that the cDNAs produced from plasmid borne elements will be distinguished from endogenous (genomic) *Ty1* elements.

Southern blotting showed that *Ty1his3-AI* elements with motif adopting G4s exhibited similar amounts of cDNA as elements not forming quadruplexes (pM2+/− and pV*Ty1*; Figure 5E). However, *Ty1his3-AI* elements having a G4 motif on template strand (pWT- and pM1−) gave rise to an additional band around 1.8 kb (Figure 5C,D,F). Such a band coincides with the length of the *AflII*-G4 fragment originating from unintegrated cDNA in which reverse transcription was stopped at G4. However, we observed no increase in intensity of this band in the case of NMM treatment nor *pif1::kanMX4* mutation. Such an observation would be expected in the case of stabilization of G4 by NMM treatment or *pif1::kanMX4* mutation. Nevertheless, the presence of shorter cDNA indicates the abortion of reverse transcription by the G4 and thus reveals the potential molecular mechanisms standing behind the inhibitory effect of G4 on *Ty1his3-AI* retrotransposition.

## 4. Discussion

The presence of G4 motifs within specific parts of LTR retrotransposons indicates that these sequences could play a role in retrotransposition regulation. We previously showed that the majority of G4 motifs are located within long terminal repeats (LTRs) where quadruplexes are close to promoters and the functional importance of G4s is strengthened by their higher number in evolutionary younger elements [9,10]. Nevertheless, other parts of LTR retrotransposons with regulatory function, namely 3′UTR, also exhibit higher G4 forming potential. We show that there are no endogenous G4 forming sequences within the yeast *Ty1his3-AI* element and hence we inserted a G4 motif into the 3′UTR. Higher regulatory potential and lower selective constraints were expected since the 3′UTR region often contains additional sequences such as extra ORF and satellites [30]. Moreover, since transcription ends inside the 3′LTR, the predicted G4s present in LTRs may actually be part of the 3′UTR as well.

Our results showed that insertion of QSs into the 3′UTR of the *Ty1his3-AI* retrotransposon impeded retrotransposition frequency. Notably, the effect of G-rich strand orientation was more important than the stability of the G4s. Although the inhibitory effect was more pronounced when G4s were formed on the template strand for transcription and potentially could block RNA polymerase, we did not observe any correlation between *Ty1his3-AI* mRNA levels and the presence of G4s. It should be noted that transcription from Gal1 promoter leads to strong overexpression compared to LTR promoter [17]. Moreover, transcription level is a poor predictor of transpositional potential due to post-transcriptional control of this process [31] and mRNA quantities do not have to correspond to altered transcription. On the other hand, elements carrying G4-forming sequences with guanines on the template strand exhibited unique bands around 1.8 kb on Southern blots suggesting the interruption of reverse transcription by G4s. Due to the strand specific nature of this band and probe design, we suggest that reverse transcription is blocked at the last stage after the second “jump” when the full length cDNA is synthesized. In any case, this lower molecular weight band is a result of plasmid borne *Ty1his3-AI* retrotransposition since it is missing in control samples where there is neither galactose induction nor plasmid carrying *Ty1his3-AI* element (Figure 5C, lines 8 and 9, respectively). We also observed an even shorter band (1.6–1.7 kb) that is probably intermediate of reverse transcription which can lead to aberrant byproducts [32]. Elements carrying less stable G4s (M1 vs. WT) showed better responsiveness to G4-stabilizing conditions In Vivo (which is in agreement with in vitro observations in physiological potassium concentration) and also produced more prominent extra bands on Southern blots. This suggests that the stability of G4s could play a crucial role for its regulatory potential, namely, less stable G4s could be more sensitive regulators than those of greater stability. In other words, the in vitro thermostability does not necessarily reflect In Vivo potential of G4 structures and further experiments with different G4s would be helpful.

Since mRNA levels and deviant products of reverse transcription did not fully explain the differences between retrotransposition frequencies of elements with or without quadruplexes, we can conclude that at least other stages of *Ty1his3-AI* life-cycle are also affected by quadruplex formation, i.e., translation, virus-like particle assembly and/or cDNA integration.

We used NMM treatment and *pif1::kanMX4* mutation strain to enhance the G4-effect on retrotransposition by G4 stabilization. Although the most suppressed retrotransposition was recorded for pWT− and pM1− in these conditions (Figure 4C), the effect of both NMM and *pif1::kanMX4* does not seem to be G4 specific. The main reason is that NMM treatment as well as *pif1::kanMX4* mutation also decreased *Ty1his3-AI* retrotransposition irrespective of G4 presence in the 3′UTR (even in both pM2+/− and pV*Ty1*). This could be explained by the fact that NMM treatment and *pif1::kanMX4* mutation exhibited lower amounts of *Ty1his3-AI* mRNA compared to untreated BY4741. However, the reduction of *Ty1his3-AI* transcript due to the absence of Pif1 helicase was not proportional to the overall retrotransposition reduction (1.7-fold vs. 3.6-fold for pV*Ty1*, respectively) which indicates that the lower mRNA levels only partially explain the reduction of retrotransposition frequency. Another reason is that both *pif1::kanMX4* and NMM also decreased the retrotransposition frequency of pM2+/− relative to pV*Ty1* (Figure 4D). Nevertheless, based on in vitro experiments, M2 sequence does not interact with NMM and the effect of M2 orientation was not prominent. Therefore, we hypothesize that the retrotransposition reduction could be caused by high GC content of M2 and/or high stability of a hairpin formed by M2 in combination with NMM treatment or *pif1::kanMX4* mutation. It could be expected that both NMM treatment and Pif1p deficiency have large scale effects on various cellular processes. Nevertheless, the G4-non-specific effect of G4 stabilization can be explained. Firstly, both WT and M1 form very stable G4 in physiological potassium concentration (>83 °C; Figure S4C) and it is questionable whether further stabilization is relevant to biological processes that take place in much lower temperatures. Secondly, since the main effect of G4 was observed during reverse transcription, Pif1 helicase was not shown to be encapsulated in VLP and hence the effect would be Pif1p independent, even though RNA–DNA hybrid is preferential substrate for this helicase [33].

To our knowledge, there is no evidence of Pif1p regulating the *Ty1his3-AI* retrotransposition and also that no studies have been performed to analyze the connection between G4 and *Ty1his3-AI* retrotransposition. Although a closely related helicase, Rrm3p (one of two Pif1 helicase family members in *S. cerevisiae*), has been studied with respect to *Ty1his3-AI* transposition [34]. Unlike Pif1p deficiency, *rrm3*Δ mutation leads to increased *Ty1his3-AI* retrotransposition and cDNA multimer insertion. These opposite effects of Rrm3p and Pif1p deficiency on *Ty1his3-AI* retrotransposition are puzzling, but several studies showed opposite effects on other processes that could be G4 dependent. Antagonistic roles have been shown in maintaining unidirectional replication fork barriers in rDNA genes, telomere erosion/lengthening, and rDNA recombination [35]. rDNA genes have high strand-specific G4 forming potential [22] and telomeric sequences have also been shown to form G4s [36]. Moreover, a number of other proteins were shown to regulate *Ty1his3-AI* retrotransposition [16], and also to bind and process G4s: notably Sgs1 helicase [37] and Xrn1 nuclease [38]. It is tempting to speculate that there is some general relationship between G4s and *Ty1his3-AI* retrotransposition. However, Pif1 is a multifuncional helicase with distinct roles in replication through non-histone proteins, lagging strand replication, replication fork convergence and double strand break repair [39]. Inhibition of all these pathways may affect *Ty1his3-AI* retrotransposition and therefore more evidence is needed.

There are a few studies that show that G4 can have both stimulatory and inhibitory effects on retrotransposition. For example, small-ligand stabilization of G-quadruplexes within the 3′UTR of human LINE-1 elements stimulated retrotransposition [40]. On the other hand, the presence of G-quadruplexes has inhibited the reverse transcription of HIV-1 [41]. Similarly, we have recently shown that maize seedlings grown in the presence of the quadruplex-stabilizing drug NMM changed the transcription of the LTR retrotransposon; having a stimulatory effect in some families and an inhibitory effect in the others [18].

## 5. Conclusions

Even though we used an artificial retrotransposon system, our results suggest that from the mechanistic point of view DNA conformation, namely four-stranded structures, may represent a challenge for the enzymes to go through. Since G4 formation is a cell cycle dependent and controlled process [42,43], these structures can represent an important factor tuning the activity of mobile genetic elements that in turn significantly shape eukaryotic genomes during evolution. The greatest advantage of DNA conformation-based regulation of transposable elements is that DNA conformation sensitively responds to environmental changes, e.g., ionic composition, enabling a better adaptation of a host species. Compared to epigenetic changes that are also reversible, G4 DNA conformation represents a faster and more flexible tool for genome response to the environment. However, the molecular mechanism by which G4 motifs modulate retrotransposon mobility, involving DNA, RNA or protein levels, still represents a challenging question for further research.

## Data Availability

Data supporting the findings of this study are available within the article and/or its supplementary materials. The raw data are available upon request.

## Supplementary Materials

The following are available at https://www.mdpi.com/article/10.3390/biology10040347/s1. Figure S1: Analysis of endogenous PQS in Ty1his3-AI retrotransposon. Figure S2: Native PAGE. Figure S3. Effect of potassium and NMM concentration on G4 formation and kinetics measured by circular dichroism (CD). Figure S4. DNA melting assay in 150 mM potassium. Table S1. Overview of oligonucleotides used in this study. Supplemental file S1: Ty1his3-AI sequence.
