# Supplementary material for: Quadruplex-Forming Motif Inserted into 3′UTR of Ty1his3-AI Retrotransposon Inhibits Retrotransposition in Yeast
Source: Biology (Basel). 2021 Apr 20;10(4):347. doi: 10.3390/biology10040347 (PMC8074290; doi:10.3390/biology10040347)
Supplement: Supplementary file 1 [file biology-10-00347-s001.zip › Supplemental materials.pdf]

Supplemental materials

Quadruplex-forming motif inserted into 3'UTR of Ty1 retrotransposon inhibits retrotransposition in yeast

Viktor Tokan[1], Jose Luis Rodriguez Lorenzo[1], Pavel Jedlicka[1], Iva Kejnovska[2], Roman Hobza[1], Eduard Kejnovsky[1]*

[1]*Department of Plant Developmental Genetics, Institute of Biophysics of the Czech Academy of Sciences, Kralovopolska 135, 61200 Brno, Czech Republic*

[2]*Department of Biophysics of Nucleic Acids, Institute of Biophysics of the Czech Academy of Sciences, Kralovopolska 135, 61200 Brno, Czech Republic*

* Author for Correspondence: Eduard Kejnovsky, Department of Plant Developmental Genetics, Institute of Biophysics of the Czech Academy of Sciences, Kralovopolska 135, CZ-612 00 Brno, Czech Republic, phone: +420-541517203, E-mail: kejnovsk@ibp.cz

Name	sequence 5´-3´	Use
URA3_F	GCGGCAGAAGAAGTAACAAAGG	qRT-PCR
URA3_R	ATCTTTGTCGCTCTTCGCAATG	
His3_F	CCTTCGTTTATCTTGCCTGCTC	
His3_R	TTTTCCACCTAGCGGATGACTC	
WT	CCCCGGGGCGGGGCGGGGCGGGGCGGGG	CD spectroscopy
M1	CCCCGGGGCGGGGCGAAGCGGGGCGGGG	
M2	CCCCGAAGCGAAGCGAAGCGAAGCGGGG	
Ty1_probe_F	TGGTGGAGGGAACATCGTTG	southern blot probe
Ty1_probe_R	ATTCCGGCTGGTCGCTAATC	
WT_top	CGGGGCGGGGCGGGGCGGGGC	plasmid construction
WT_bottom	GCCCCGCCCCGCCCCGCCCCG	
M1_top	CGGGGCGGGGCGAAGCGGGGC	
M1_bottom	GCCCCGCTTCGCCCCGCCCCG	
M2_top	CGAAGCGAAGCGAAGCGAAGC	
M2_bottom	GCTTCGCTTCGCTTCGCTTCG	

Table S1. Overview of oligonucleotides used in this study

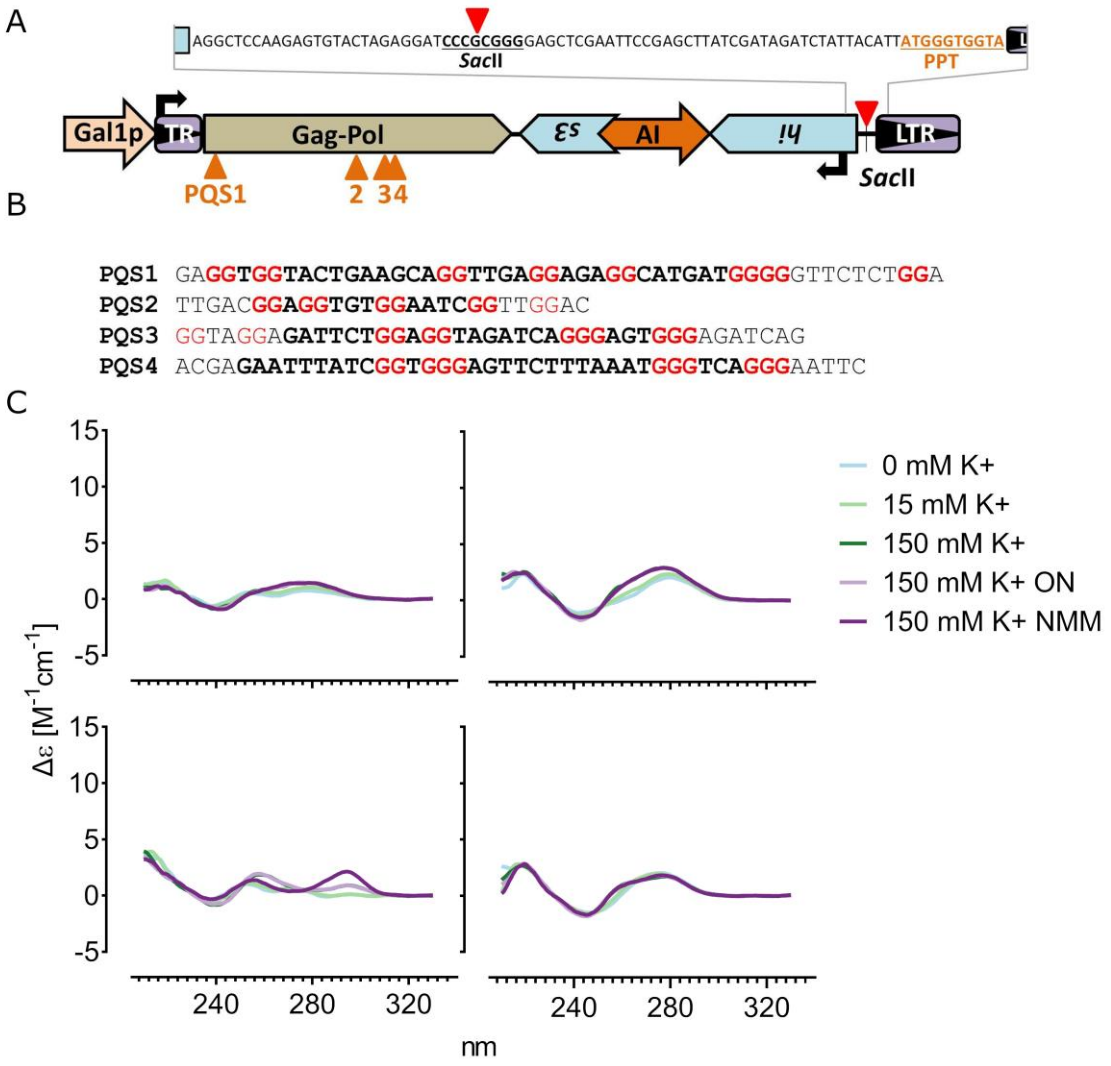

Figure S1. Analysis of endogenous PQS in *Ty1his3-AI* retrotransposon.

(A) A schematic localisation of predicted potential quadruplex forming sequences (PQS; orange triangles) within *Ty1his3-AI* retrotransposon. G-rich strand of all predicted sequences is template strand for transcription. Red triangles show the cloning *SacII* site where sequences of interest were inserted. The polypurine tract (PPT) is also highlighted. (**B)** Oligonucleotides used for CD spectroscopy measurement containing both predicted sequences (bold) and surrounding sequences. Guanines possibly forming G4 are red. (**C)** CD spectra obtained in increasing potassium concentration at 22 °C. After overnight incubation (ON) in 150mM K^+ samples were also measured with the addition of 7,5 µM NMM to force G4 formation.

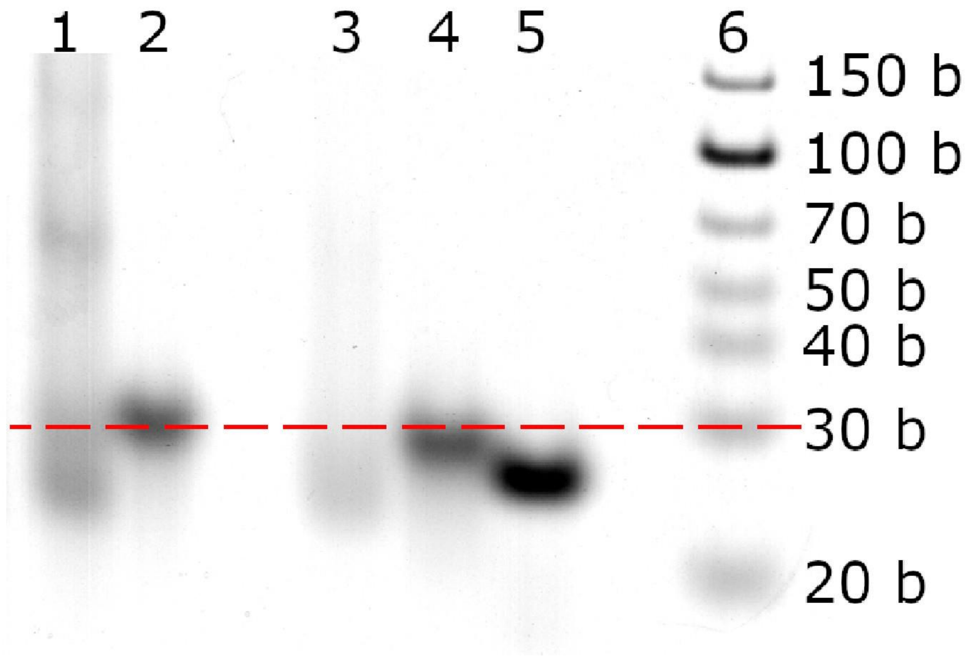

Figure S2. Native PAGE

Annealed oligonucleotides WT and M1 (lines 1 and 2), oligonucleotides WT, M1 and M2 without annealing (lines 3-5), size marker (line 6). All tested oligonucleotides adopted monomolecular organization but WT also formed a bimolecular fraction after annealing in 150 mM potassium.

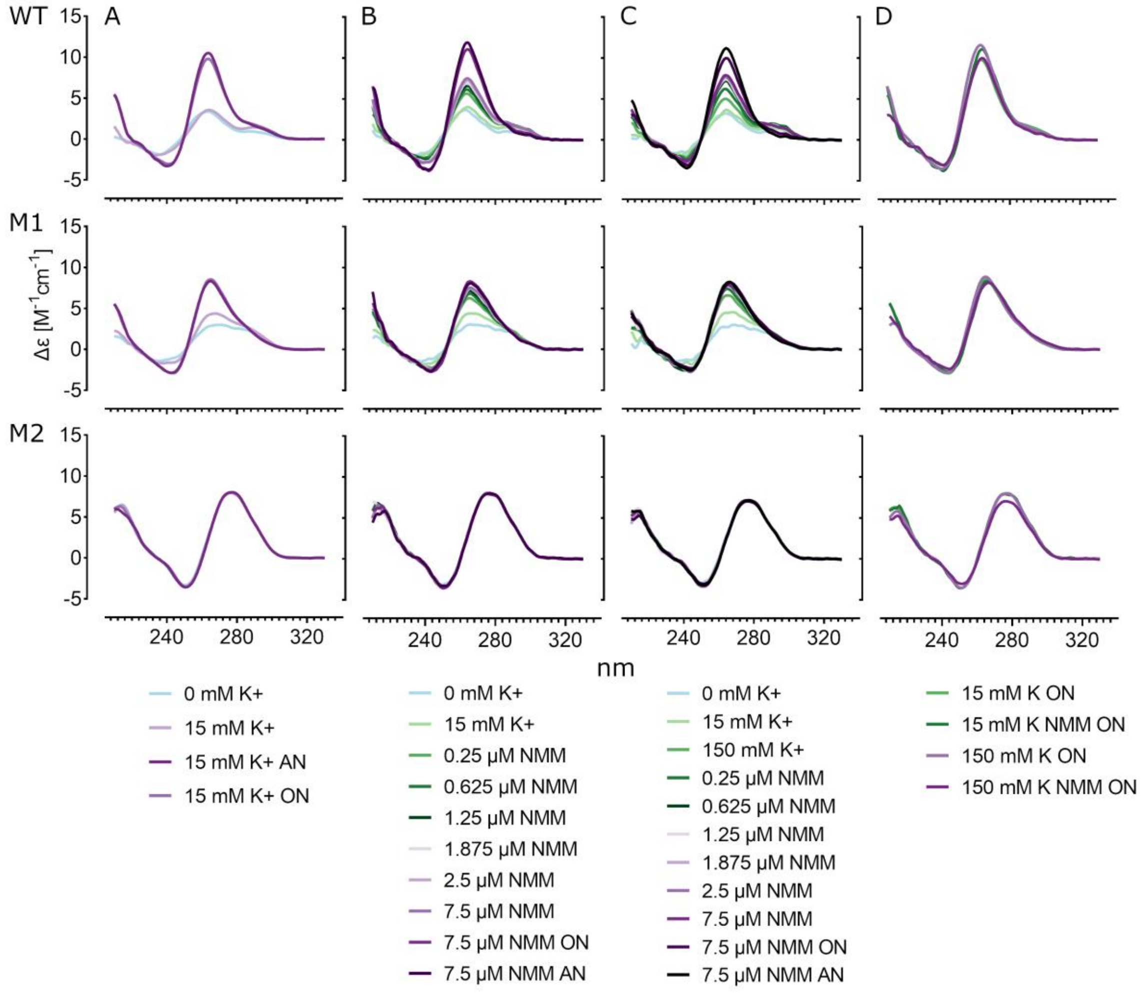

Figure S3. Effect of potassium and NMM concentration on G4 formation and kinetics measured by circular dichroism (CD). (A) CD obtained in final 15mM potassium. **(B)** Increasing NMM concentration in presence of 15mM potassium ions shows acceleration of G4 formation. **(C)** Effect of an increasing NMM concentration on the ellipticity in presence of 150mM potassium ions showing similar results as in 15mM potassium. **(D)** Comparison of overnight incubated samples in both 15mM and 150mM potassium as well as with and without NMM shows negligible differences which suggest that 15mM potassium is fully sufficient to induce G4 formation and NMM have no effect on G4 topology and orderliness. Note that M2 spectra remain unaffected by both NMM and potassium concentration. ON - overnight, AN - annealed.

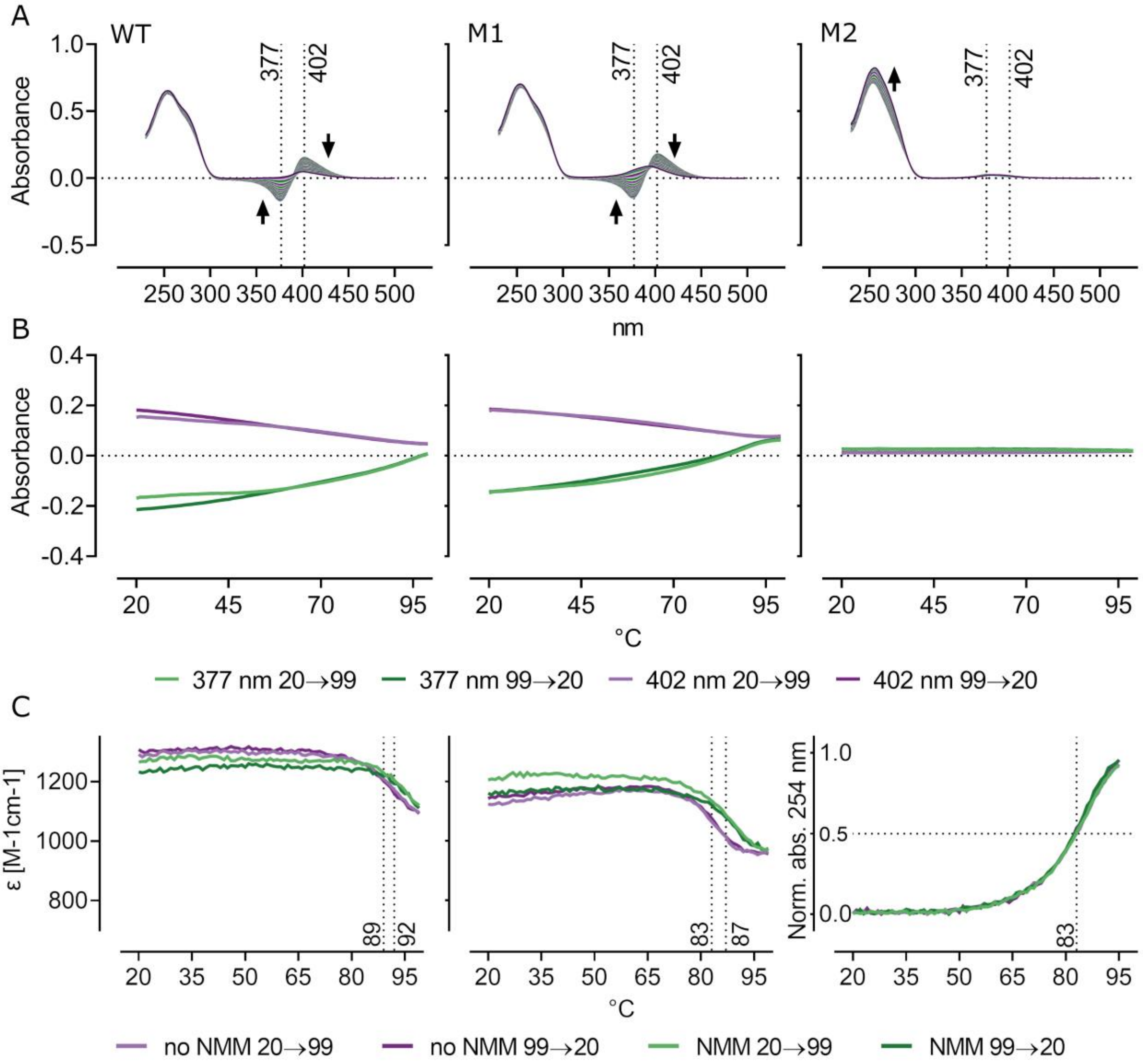

Figure S4. DNA melting assay in 150 mM potassium

(A) Full absorption spectra obtained during heating samples from 20 to 99°C in 150 mM potassium with 7.5 µM NMM present typical peaks at 377 and 402 nm of free and G4-bound NMM respectively. Black arrows indicate peak development with increasing temperature. **(B)** Absorbance at 377 and 402 nm plotted against temperature revealing a disruption of NMM-G4 interaction by increasing temperature and reassociation when cooling down. **(C)** Comparison of melting curves at 297 nm in 150 mM potassium shows a shift towards a higher temperature due to NMM presence. Indicated Tm are rough estimates calculated from normalised data. Melting curves for M2 are calculated as normalised absorbance at 254 nm.

Supplemental file 1. *Ty1his3-AI* sequence

>Ty1his3-AI
ACGGATTAGAAGCCGCCGAGCGGGCGACAGCCCTCCGACGGAAGACTCTCCTCCGTGCGTCCTCGTCT
TCACCGGTCGCGTTCCTGAAACGCAGATGTGCCTCGCGCCGCACTGCTCCGAACAATAAAGATTCTAC
AATACTAGCTTTTATGGTTATGAAGAGGAAAAATTGGCAGTAACCTGGCCCCACAAACCTTCAAATTA
ACGAATCAAATTAACAACCATAGGATGATAATGCGATTAGTTTTTTAGCCTTATTTCTGGGGTAATTA
ATCAGCGAAGCGATGATTTTTGATCTATTAACAGATATATAAATGGAAAAGCTGCATAACCACTTTAA
CTAATACTTTCAACATTTTCGGTTTGTATTACTTCTTATTCCTCTACCGCCTCGAGGAGAACTTCTAG
TATATTCTGTATACCTAATATTATAGCCTTTATCAACAATGGAATCCCAACAATTATCTCAACATTCA
CCCAATTCTCATGGTAGCGCCTGTGCTTCGGTTACTTCTAAGGAAGTCCACACAAATCAAGATCCGTT
AGACGTTTCAGCTTCCAAAACAGAAGAATGTGAGAAGGCTTCCACTAAGGCTAACTCTCAACAGACAA
CAACACCTGCTTCATCAGCTGTTCCAGAGAACCCCCATCATGCCTCTCCTCAACCTGCTTCAGTACCA
CCTCCACAGAATGGGCCGTACCCACAGCAGTGCATGATGACCCAAAACCAAGCCAATCCATCTGGTTG
GTCATTTTACGGACACCCATCTATGATTCCGTATACACCTTATCAAATGTCGCCTATGTACTTTCCAC
CTGGGCCACAATCACAGTTTCCGCAGTATCCATCATCAGTTGGAACGCCTCTGAGCACTCCATCACCT
GAGTCAGGTAATACATTTACTGATTCATCCTCAGCGGACTCTGATATGACATCCACTAAAAAATATGT
CAGACCACCACCAATGTTAACCTCACCTAATGACTTTCCAAATTGGGTTAAAACATACATCAAATTTT
TACAAAACTCGAATCTCGGTGGTATTATTCCGACAGTAAACGGAAAACCCGTACGTCAGATCACTGAT
GATGAACTCACCTTCTTGTATAACACTTTTCAAATATTTGCTCCCTCTCAATTCCTACCTACCTGGGT
CAAAGACATCCTATCCGTTGATTATACGGATATCATGAAAATTCTTTCCAAAAGTATTGAAAAAATGC
AATCTGATACCCAAGAGGCAAACGACATTGTGACCCTGGCAAATTTGCAATATAATGGCAGTACACCT
GCAGATGCATTTGAAACAAAAGTCACAAACATTATCGACAGACTGAACAATAATGGCATTCATATCAA
TAACAAGGTCGCATGCCAATTAATTATGAGAGGTCTATCTGGCGAATATAAATTTTTACGCTACACAC
GTCATCGACATCTAAATATGACAGTCGCTGAACTGTTCTTAGATATCCATGCTATTTATGAAGAACAA
CAGGGATCGAGAAACAGTAAACCTAATTACAGGAGAAATCCGAGTGATGAGAAGAATGATTCTCGCAG
CTATACGAATACAACCAAACCCAAAGTTATAGCTCGGAATCCTCAAAAAACAAATAATTCGAAATCGA
AAACAGCCAGGGCTCACAATGTATCCACATCTAATAACTCTCCCAGCACGGACAACGATTCCATCAGT
AAATCAACTACTGAACCGATTCAATTGAACAATAAGCACGACCTTCATCTTAGGCCAGAAACTTACTG
AATCTACAGTAAATCATACTAATCATTCTGATGATGAACTCCCTGGACACCTCCTTCTCGATTCAGGA
GCATCACGAACCCTTATAAGATCTGCTCATCACATACACTCAGCATCATCTAATCCTGACATAAACGT
AGTTGATGCTCAAAAAAGAAATATACCAATTAACGCTATTGGTGACCTACAATTTCACTTCCAGGACA
ACACCAAAACATCAATAAAGGTATTGCACACTCCTAACATAGCCTATGACTTACTCAGTTTGAATGAA
TTGGCTGCAGTAGATATCACAGCATGCTTTACCAAAAACGTCTTAGAACGGTCTGACGGCACTGTACT
TGCACCTATCGTAAAATATGGAGACTTTTACTGGGTATCTAAAAAGTACTTGCTTCCATCAAATATCT
CCGTACCCACCATCAATAATGTCCATACAAGTGAAAGTACACGCAAATATCCTTATCCTTTCATTCAT
CGAATGCTTGCGCATGCCAATGCACAGACAATTCGATACTCACTTAAAAATAACACCATCACGTATTT
TAACGAATCAGATGTCGACTGGTCTAGTGCTATTGACTATCAATGTCCTGATTGTTTAATCGGCAAAA
GCACCAAACACAGACATATCAAAGGTTCACGACTAAAATACCAAAATTCATACGAACCCTTTCAATAC
CTACATACTGACATATTTGGTCCAGTTCACAACCTACCAAATAGTGCACCATCCTATTTCATCTCATT
TACTGATGAGACAACAAAATTCCGTTGGGTTTATCCATTACACGACCGTCGCGAGGACTCTATCCTCG
ATGTTTTTACTACGATACTAGCTTTTATTAAAAACCAGTTTCAGGCCAGTGTCTTGGTTATACAAATG
GACCGTGGTTCTGAGTATACTAACAGAACTCTCCATAAATTCCTTGAAAAAAATGGTATAACTCCATG
CTATACAACCACAGCGGATTCCCGAGCACATGGAGTCGCTGAACGGCTAAACCGTACCTTATTAGATG
ACTGCCGTACTCAACTGCAATGTAGTGGTTTACCGAACCATTTATGGTTCTCTGCAATCGAATTTTCT
ACTATTGTGAGAAATTCACTAGCTTCACCTAAAAGCAAAAAATCTGCAAGACAACATGCTGGCTTGGC
AGGACTTGATATCAGTACTTTGTTACCTTTCGGTCAACCTGTTATCGTCAATGATCACAACCCTAACT
CCAAAATACATCCTCGTGGCATCCCAGGCTACGCTCTACATCCGTCTCGAAACTCTTATGGATATATC
ATCTATCTTCCATCCTTAAAGAAGACAGTAGATACAACTAACTATGTTATTCTTCAGGGCAAGGAATC

CAGATTAGATCAATTCAATTACGACGCACTCACTTTCGATGAAGACTTAAACCGTTTAACTGCTTCAT
ATCATTCGTTCATTGCGTCAAATGAGATCCAAGAATCCAATGATCTTAACATAGAATCTGACCATGAC
TTCCAATCCGACATTGAACTACATCCTGAGCAACCGAGAAATGTCCTTTCAAAAGCTGTGAGTCCAAC
CGATTCCACACCTCCGTCAACTCATACTGAAGATTCGAAACGTGTTTCTAAAACCAATATTCGCGCAC
CCAGAGAAGTTGACCCCAACATATCTGAATCTAATATTCTTCCATCAAAGAAGAGATCTAGCACCCCC
CAAATTTCCAATATCGAGAGTACCGGTTCGGGTGGTATGCATAAATTAAATGTTCCTTTACTTGCTCC
CATGTCCCAATCTAACACACATGAGTCGTCGCACGCCAGTAAATCTAAAGATTTCAGACACTCAGACT
CGTACAGTGAAAATGAGACTAATCATACAAACGTACCAATATCCAGTACGGGTGGTACCAACAACAAA
ACTGTTCCGCAGATAAGTGACCAAGAGACTGAGAAAAGGATTATACACCGTTCACCTTCAATTGATGC
TTCTCCACCGGAAAATAATTCATCGCACAATATTGTTCCTATCAAAACGCCAACTACTGTTTCTGAAC
AGAATACCGAGGAATCTATCATCGCTGATCTCCCACTCCCTGATCTACCTCCAGAATCTCCTACCGAA
TTCCCTGACCCATTTAAAGAACTCCCACCGATAAATTCTCGTCAAACTAATTCCAGTTTGGGTGGTAT
TGGTGACTCTAATGCCTATACTACTATCAACAGTAAGAAAAGATCATTAGAAGATAATGAAACTGAAA
TTAAGGTATCACGAGACACATGGAATACTAAGAATATGCGTAGTTTAGAACCTCCGAGATCGAAGAAA
CGAATTCACCTGATTGCAGCTGTAAAAGCAGTAAAATCAATCAAACCAATACGGACAACCTTACGATA
CGATGAGGCAATCACCTATAATAAAGATATTAAAGAAAAAGAAAAATATATCGAGGCATACCACAAAG
AAGTCAATCAACTGTTGAAGATGAAAACTTGGGACACTGACGAATATTATGACAGAAAAGAAATAGAC
CCTAAAAGAGTAATAAACTCAATGTTTATCTTCAACAAGAAACGTGACGGTACTCATAAAGCTAGATT
TGTTGCAAGAGGTGATATTCAGCATCCTGACACTTACGACTCAGGCATGCAATCCAATACCGTACATC
ACTATGCATTAATGACATCCCTGTCACTTGCATTAGACAATAACTACTATATTACACAATTAGACATA
TCTTCGGCATATTTGTATGCAGACATCAAAGAAGAATTATACATAAGACCTCCACCACATTTAGGAAT
GAATGATAAGTTGATACGTTTGAAGAAATCACTTTATGGATTGAAACAAAGTGGAGCGAACTGGTACG
AAACTATCAAATCATACCTGATACAACAATGTGGTATGGAAGAAGTTCGTGGATGGTCATGCGTATTT
AAAAACAGTCAAGTGACAATTTGTTTATTCGTAGATGATATGGTATTGTTTAGCAAAAATCTAAATTC
AAACAAAAGAATTATAGAGAAGCTTAAGATGCAATACGACACCAAGATTATAAATCTAGGCGAAAGTG
ATGAGGAAATTCAATATGACATACTTGGCTTAGAAATCAAATATCAAAGAGGTAAATACATGAAATTA
GGTATGGAAAACTCATTAACTGAGAAAATACCCAAATTAAACGTACCTTTGAATCCAAAAGGAAGAAA
ACTTAGCGCTCCAGGTCAACCAGGTCTTTATATAGACCAGGATGAACTAGAAATAGATGAAGATGAAT
ACAAAGAGAAGGTACATGAAATGCAAAAGTTGATTGGTCTAGCTTCATATGTTGGATATAAATTTAGA
TTTGACTTACTATACTACATCAACACACTTGCTCAACATATACTATTCCCCTCTAGGCAAGTTTTAGA
CATGACATATGAGTTGATACAATTCATGTGGGACACTAGAGATAAACAACTGATATGGCACAAAAACA
AACCTACCGAGCCAGATAATAAACTAGTCGCAATAAGTGATGCTTCGTATGGCAACCAACCGTATTAT
AAATCACAAATTGGCAACATATATTTACTTAATGGAAAGGTAATTGGAGGAAAGTCCACCAAGGCTTC
ATTAACATGTACTTCAACTACGGAAGCAGAAATACACGCGATAAGTGAATCTGTCCCATTATTAAATA
ATCTAAGTTACCTGATACAAGAACTTAACAAGAAACCAATTATTAAAGGCTTACTTACTGATAGTAGA
TCAACGATCAGTATAATTAAGTCTACAAATGAAGAGAAATTTAGAAACAGATTTTTTGGCACAAAGGC
AATGAGACTTAGAGATGAAGTATCAGGTAATAATTTATACGTATACTACATCGAGACCAAGAAGAACA
TTGCTGATGTGATGACAAAACCTCTTCCGATAAAAACATTTAAACTATTAACTAACAAATGGATTCAT
TAGATCTATCGATAAGCTTCTGCAGCTTTAAATAATCGGTGTCACTACATAAGAACACCTTTGGTGGA
GGGAACATCGTTGGTACCATTGGGCGAGGTGGCTTCTCTTATGGCAACCGCAAGAGCCTTGAACGCAC
TCTCACTACGGTGATGATCATTCTTGCCTCGCAGACAATCAACGTGGAGGGTAATTCTGCTAGCCTCT
GCAAAGCTTTCAAGAAAATGCGGGATCATCTCGCAAGAGAGATCTCCTACTTTCTCCCTTTGCAAACC
AAGTTCGACAACTGCGTACGGCCTGTTCGAAAGATCTACCACCGCTCTGGAAAGTGCCTCATCCAAAG
GCGCAAATCCTGATCCAAACCTTTTTACTCCACGCACGGCCCCTAGGGCCTCTTTAAAAGCTTGACCG
AGAGCAATCCCGCAGTCTTCAGTGGTGTGATGGTCGTCTATGTGTAAGTCACCAATGCACTCAACGAT
TAGCGACCAGCCGGAATGCTTGGGTATGTTAATATGGACTAAAGGAGGCTTTTCTGCAGGTCGACTCT
AGAGGATCCCCGGGTACCGAGCTCGAATTTTTACTAACAAATGGTATTATTTATAACAGCCAGAGCAT
GTATCATATGGTCCAGAAACCCTATACCTGTGTGGACGTTAATCACTTGCGATTGTGTGGCCTGTTCT

GCTACTGCTTCTGCCTCTTTTTCTGGGAAGATCGAGTGCTCTATCGCTAGGGGACCACCCTTTAAAGA
GATCGCAATCTGAATCTTGGTTTCATTTGTAATACGCTTTACTAGGGCTTTCTGCTCTGTCATCTTTG
CCTTCGTTTATCTTGCCTGCTCATTTTTTAGTATATTCTTCGAAGAAATCACATTACTTTATATAATG
TATAATTCATTATGTGATAATGCCAATCGCTAAGAAAAAAAAAAGAGTCATCCGCTAGGTGGAAAAAAA
AAAATGAAAATCATTACCGAGGCATAAAAAAAATATAGAGTGTACTAGAGGCTCCAAGAGTGTACTAGA
GGATCCCGCGGGGAGCTCGAATTCCGAGCTTATCGATAGATCTATTACATTATGGGTGGTATGTTGGA
ATAGAAATCAACTATCATCTACTAACTAGTATTTACATTACTAGTATATTATCATATACGGTGTTAGA
AGATGACGCAAATGATGAGAAATAGTCATCTAAATTAGTGGAAGCTGAAACGCAAGGATTGATAATGT
AATAGGATCAATGAATATAAACATATAAAATGATGATAATAATATTTATAGAATTGTGTAGAATTGCA
GATTCCCTTTTATGGATTCCTAAATCCTTGAGGAGAACTTCTAGTATATTCTGTATACCTAATATTAT
AGCCTTTATCAACAATGGAATCCCAACAATTATCTCAACATTCACCCATTTCTCA
