# Supplementary material for: Quadruplex-Forming Motif Inserted into 3′UTR of Ty1his3-AI Retrotransposon Inhibits Retrotransposition in Yeast
Source: Biology (Basel). 2021 Apr 20;10(4):347. doi: 10.3390/biology10040347 (PMC8074290; doi:10.3390/biology10040347)
Supplement: Supplementary file 1 [file biology-10-00347-s001.zip › Table S1 v2.pdf]

Name	sequence 5´-3´	Use
**URA3_F**	GCGGCAGAAGAAGTAACAAAGG	qRT-PCR
**URA3_R**	ATCTTTGTCGCTCTTCGCAATG	
**His3_F**	CCTTCGTTTATCTTGCCTGCTC	
**His3_R**	TTTTCCACCTAGCGGATGACTC	
**WT**	CCCCGGGGCGGGGCGGGGCGGGGCGGGG	CD spectroscopy
**M1**	CCCCGGGGCGGGGCGAAGCGGGGCGGGG	
**M2**	CCCCGAAGCGAAGCGAAGCGAAGCGGGG	
**Ty1_probe_F**	TGGTGGAGGGAACATCGTTG	southern blot probe
**Ty1_probe_R**	ATTCCGGCTGGTCGCTAATC	
**WT_top**	CGGGGCGGGGCGGGGCGGGGC	plasmid construction
**WT_bottom**	GCCCCGCCCCGCCCCGCCCCG	
**M1_top**	CGGGGCGGGGCGAAGCGGGGC	
**M1_bottom**	GCCCCGCTTCGCCCCGCCCCG	
**M2_top**	CGAAGCGAAGCGAAGCGAAGC	
**M2_bottom**	GCTTCGCTTCGCTTCGCTTCG	
