# Supplementary material for: Quadruplex-Forming Motif Inserted into 3′UTR of Ty1his3-AI Retrotransposon Inhibits Retrotransposition in Yeast
Source: Biology (Basel). 2021 Apr 20;10(4):347. doi: 10.3390/biology10040347 (PMC8074290; doi:10.3390/biology10040347)
Supplement: Supplementary file 1 [file biology-10-00347-s001.zip › File S1.pdf]

>Ty1his3-AI
ACGGATTAGAAGCCGCCGAGCGGGCGACAGCCCTCCGACGGAAGACTCTCCTCCGTGCGTCCTCGTCTTCACC
GGTCGCGTTCCTGAAACGCAGATGTGCCTCGCGCCGCACTGCTCCGAACAATAAAGATTCTACAATACTAGCT
TTTATGGTTATGAAGAGGAAAAATTGGCAGTAACCTGGCCCCACAAACCTTCAAATTAACGAATCAAATTAAC
AACCATAGGATGATAATGCGATTAGTTTTTTAGCCTTATTTCTGGGGTAATTAATCAGCGAAGCGATGATTTT
TGATCTATTAACAGATATATAAATGGAAAAGCTGCATAACCACTTTAACTAATACTTTCAACATTTTCGGTTT
GTATTACTTCTTATTCCTCTACCGCCTCGAGGAGAACTTCTAGTATATTCTGTATACCTAATATTATAGCCTT
TATCAACAATGGAATCCCAACAATTATCTCAACATTCACCCAATTCTCATGGTAGCGCCTGTGCTTCGGTTAC
TTCTAAGGAAGTCCACACAAATCAAGATCCGTTAGACGTTTCAGCTTCCAAAACAGAAGAATGTGAGAAGGCT
TCCACTAAGGCTAACTCTCAACAGACAACAACACCTGCTTCATCAGCTGTTCCAGAGAACCCCCATCATGCCT
CTCCTCAACCTGCTTCAGTACCACCTCCACAGAATGGGCCGTACCCACAGCAGTGCATGATGACCCAAAACCA
AGCCAATCCATCTGGTTGGTCATTTTACGGACACCCATCTATGATTCCGTATACACCTTATCAAATGTCGCCT
ATGTACTTTCCACCTGGGCCACAATCACAGTTTCCGCAGTATCCATCATCAGTTGGAACGCCTCTGAGCACTC
CATCACCTGAGTCAGGTAATACATTTACTGATTCATCCTCAGCGGACTCTGATATGACATCCACTAAAAAATA
TGTCAGACCACCACCAATGTTAACCTCACCTAATGACTTTCCAAATTGGGTTAAAACATACATCAAATTTTTA
CAAAACTCGAATCTCGGTGGTATTATTCCGACAGTAAACGGAAAACCCGTACGTCAGATCACTGATGATGAAC
TCACCTTCTTGTATAACACTTTTCAAATATTTGCTCCCTCTCAATTCCTACCTACCTGGGTCAAAGACATCCT
ATCCGTTGATTATACGGATATCATGAAAATTCTTTCCAAAAGTATTGAAAAAATGCAATCTGATACCCAAGAG
GCAAACGACATTGTGACCCTGGCAAATTTGCAATATAATGGCAGTACACCTGCAGATGCATTTGAAACAAAAG
TCACAAACATTATCGACAGACTGAACAATAATGGCATTCATATCAATAACAAGGTCGCATGCCAATTAATTAT
GAGAGGTCTATCTGGCGAATATAAATTTTTACGCTACACACGTCATCGACATCTAAATATGACAGTCGCTGAA
CTGTTCTTAGATATCCATGCTATTTATGAAGAACAACAGGGATCGAGAAACAGTAAACCTAATTACAGGAGAA
ATCCGAGTGATGAGAAGAATGATTCTCGCAGCTATACGAATACAACCAAACCCAAAGTTATAGCTCGGAATCC
TCAAAAAACAAATAATTCGAAATCGAAAACAGCCAGGGCTCACAATGTATCCACATCTAATAACTCTCCCAGC
ACGGACAACGATTCCATCAGTAAATCAACTACTGAACCGATTCAATTGAACAATAAGCACGACCTTCATCTTA
GGCCAGAAACTTACTGAATCTACAGTAAATCATACTAATCATTCTGATGATGAACTCCCTGGACACCTCCTTC
TCGATTCAGGAGCATCACGAACCCTTATAAGATCTGCTCATCACATACACTCAGCATCATCTAATCCTGACAT
AAACGTAGTTGATGCTCAAAAAAGAAATATACCAATTAACGCTATTGGTGACCTACAATTTCACTTCCAGGAC
AACACCAAAACATCAATAAAGGTATTGCACACTCCTAACATAGCCTATGACTTACTCAGTTTGAATGAATTGG
CTGCAGTAGATATCACAGCATGCTTTACCAAAAACGTCTTAGAACGGTCTGACGGCACTGTACTTGCACCTAT
CGTAAAATATGGAGACTTTTACTGGGTATCTAAAAAGTACTTGCTTCCATCAAATATCTCCGTACCCACCATC
AATAATGTCCATACAAGTGAAAGTACACGCAAATATCCTTATCCTTTCATTCATCGAATGCTTGCGCATGCCA
ATGCACAGACAATTCGATACTCACTTAAAAATAACACCATCACGTATTTTAACGAATCAGATGTCGACTGGTC
TAGTGCTATTGACTATCAATGTCCTGATTGTTTAATCGGCAAAAGCACCAAACACAGACATATCAAAGGTTCA
CGACTAAAATACCAAAATTCATACGAACCCTTTCAATACCTACATACTGACATATTTGGTCCAGTTCACAACC
TACCAAATAGTGCACCATCCTATTTCATCTCATTTACTGATGAGACAACAAAATTCCGTTGGGTTTATCCATT
ACACGACCGTCGCGAGGACTCTATCCTCGATGTTTTTACTACGATACTAGCTTTTATTAAAAACCAGTTTCAG
GCCAGTGTCTTGGTTATACAAATGGACCGTGGTTCTGAGTATACTAACAGAACTCTCCATAAATTCCTTGAAA
AAAATGGTATAACTCCATGCTATACAACCACAGCGGATTCCCGAGCACATGGAGTCGCTGAACGGCTAAACCG
TACCTTATTAGATGACTGCCGTACTCAACTGCAATGTAGTGGTTTACCGAACCATTTATGGTTCTCTGCAATC
GAATTTTCTACTATTGTGAGAAATTCACTAGCTTCACCTAAAAGCAAAAAATCTGCAAGACAACATGCTGGCT
TGGCAGGACTTGATATCAGTACTTTGTTACCTTTCGGTCAACCTGTTATCGTCAATGATCACAACCCTAACTC
CAAAATACATCCTCGTGGCATCCCAGGCTACGCTCTACATCCGTCTCGAAACTCTTATGGATATATCATCTAT
CTTCCATCCTTAAAGAAGACAGTAGATACAACTAACTATGTTATTCTTCAGGGCAAGGAATCCAGATTAGATC
AATTCAATTACGACGCACTCACTTTCGATGAAGACTTAAACCGTTTAACTGCTTCATATCATTCGTTCATTGC
GTCAAATGAGATCCAAGAATCCAATGATCTTAACATAGAATCTGACCATGACTTCCAATCCGACATTGAACTA
CATCCTGAGCAACCGAGAAATGTCCTTTCAAAAGCTGTGAGTCCAACCGATTCCACACCTCCGTCAACTCATA
CTGAAGATTCGAAACGTGTTTCTAAAACCAATATTCGCGCACCCAGAGAAGTTGACCCCAACATATCTGAATC
TAATATTCTTCCATCAAAGAAGAGATCTAGCACCCCCCAAATTTCCAATATCGAGAGTACCGGTTCGGGTGGT
ATGCATAAATTAAATGTTCCTTTACTTGCTCCCATGTCCCAATCTAACACACATGAGTCGTCGCACGCCAGTA
AATCTAAAGATTTCAGACACTCAGACTCGTACAGTGAAAATGAGACTAATCATACAAACGTACCAATATCCAG
TACGGGTGGTACCAACAACAAAACTGTTCCGCAGATAAGTGACCAAGAGACTGAGAAAAGGATTATACACCGT
TCACCTTCAATTGATGCTTCTCCACCGGAAAATAATTCATCGCACAATATTGTTCCTATCAAAACGCCAACTA
CTGTTTCTGAACAGAATACCGAGGAATCTATCATCGCTGATCTCCCACTCCCTGATCTACCTCCAGAATCTCC
TACCGAATTCCCTGACCCATTTAAAGAACTCCCACCGATAAATTCTCGTCAAACTAATTCCAGTTTGGGTGGT
ATTGGTGACTCTAATGCCTATACTACTATCAACAGTAAGAAAAGATCATTAGAAGATAATGAAACTGAAATTA
AGGTATCACGAGACACATGGAATACTAAGAATATGCGTAGTTTAGAACCTCCGAGATCGAAGAAACGAATTCA
CCTGATTGCAGCTGTAAAAGCAGTAAAATCAATCAAACCAATACGGACAACCTTACGATACGATGAGGCAATC

ACCTATAATAAAGATATTAAAGAAAAAGAAAAATATATCGAGGCATACCACAAAGAAGTCAATCAACTGTTGA
AGATGAAAACTTGGGACACTGACGAATATTATGACAGAAAAGAAATAGACCCTAAAAGAGTAATAAACTCAAT
GTTTATCTTCAACAAGAAACGTGACGGTACTCATAAAGCTAGATTTGTTGCAAGAGGTGATATTCAGCATCCT
GACACTTACGACTCAGGCATGCAATCCAATACCGTACATCACTATGCATTAATGACATCCCTGTCACTTGCAT
TAGACAATAACTACTATATTACACAATTAGACATATCTTCGGCATATTTGTATGCAGACATCAAAGAAGAATT
ATACATAAGACCTCCACCACATTTAGGAATGAATGATAAGTTGATACGTTTGAAGAAATCACTTTATGGATTG
AAACAAAGTGGAGCGAACTGGTACGAAACTATCAAATCATACCTGATACAACAATGTGGTATGGAAGAAGTTC
GTGGATGGTCATGCGTATTTAAAAACAGTCAAGTGACAATTTGTTTATTCGTAGATGATATGGTATTGTTTAG
CAAAAATCTAAATTCAAACAAAAGAATTATAGAGAAGCTTAAGATGCAATACGACACCAAGATTATAAATCTA
GGCGAAAGTGATGAGGAAATTCAATATGACATACTTGGCTTAGAAATCAAATATCAAAGAGGTAAATACATGA
AATTAGGTATGGAAAACTCATTAACTGAGAAAATACCCAAATTAAACGTACCTTTGAATCCAAAAGGAAGAAA
ACTTAGCGCTCCAGGTCAACCAGGTCTTTATATAGACCAGGATGAACTAGAAATAGATGAAGATGAATACAAA
GAGAAGGTACATGAAATGCAAAAGTTGATTGGTCTAGCTTCATATGTTGGATATAAATTTAGATTTGACTTAC
TATACTACATCAACACACTTGCTCAACATATACTATTCCCCTCTAGGCAAGTTTTAGACATGACATATGAGTT
GATACAATTCATGTGGGACACTAGAGATAAACAACTGATATGGCACAAAAACAAACCTACCGAGCCAGATAAT
AAACTAGTCGCAATAAGTGATGCTTCGTATGGCAACCAACCGTATTATAAATCACAAATTGGCAACATATATT
TACTTAATGGAAAGGTAATTGGAGGAAAGTCCACCAAGGCTTCATTAACATGTACTTCAACTACGGAAGCAGA
AATACACGCGATAAGTGAATCTGTCCCATTATTAAATAATCTAAGTTACCTGATACAAGAACTTAACAAGAAA
CCAATTATTAAAGGCTTACTTACTGATAGTAGATCAACGATCAGTATAATTAAGTCTACAAATGAAGAGAAAT
TTAGAAACAGATTTTTTGGCACAAAGGCAATGAGACTTAGAGATGAAGTATCAGGTAATAATTTATACGTATA
CTACATCGAGACCAAGAAGAACATTGCTGATGTGATGACAAAACCTCTTCCGATAAAAACATTTAAACTATTA
ACTAACAAATGGATTCATTAGATCTATCGATAAGCTTCTGCAGCTTTAAATAATCGGTGTCACTACATAAGAA
CACCTTTGGTGGAGGGAACATCGTTGGTACCATTGGGCGAGGTGGCTTCTCTTATGGCAACCGCAAGAGCCTT
GAACGCACTCTCACTACGGTGATGATCATTCTTGCCTCGCAGACAATCAACGTGGAGGGTAATTCTGCTAGCC
TCTGCAAAGCTTTCAAGAAAATGCGGGATCATCTCGCAAGAGAGATCTCCTACTTTCTCCCTTTGCAAACCAA
GTTCGACAACTGCGTACGGCCTGTTCGAAAGATCTACCACCGCTCTGGAAAGTGCCTCATCCAAAGGCGCAAA
TCCTGATCCAAACCTTTTTACTCCACGCACGGCCCCTAGGGCCTCTTTAAAAGCTTGACCGAGAGCAATCCCG
CAGTCTTCAGTGGTGTGATGGTCGTCTATGTGTAAGTCACCAATGCACTCAACGATTAGCGACCAGCCGGAAT
GCTTGGGTATGTTAATATGGACTAAAGGAGGCTTTTCTGCAGGTCGACTCTAGAGGATCCCCGGGTACCGAGC
TCGAATTTTTACTAACAAATGGTATTATTTATAACAGCCAGAGCATGTATCATATGGTCCAGAAACCCTATAC
CTGTGTGGACGTTAATCACTTGCGATTGTGTGGCCTGTTCTGCTACTGCTTCTGCCTCTTTTTCTGGGAAGAT
CGAGTGCTCTATCGCTAGGGGACCACCCTTTAAAGAGATCGCAATCTGAATCTTGGTTTCATTTGTAATACGC
TTTACTAGGGCTTTCTGCTCTGTCATCTTTGCCTTCGTTTATCTTGCCTGCTCATTTTTTAGTATATTCTTCG
AAGAAATCACATTACTTTATATAATGTATAATTCATTATGTGATAATGCCAATCGCTAAGAAAAAAAAAGAGT
CATCCGCTAGGTGGAAAAAAAAAAAATGAAAATCATTACCGAGGCATAAAAAAATATAGAGTGTACTAGAGGCT
CCAAGAGTGTACTAGAGGATCCCGCGGGGAGCTCGAATTCCGAGCTTATCGATAGATCTATTACATTATGGGT
GGTATGTTGGAATAGAAATCAACTATCATCTACTAACTAGTATTTACATTACTAGTATATTATCATATACGGT
GTTAGAAGATGACGCAAATGATGAGAAATAGTCATCTAAATTAGTGGAAGCTGAAACGCAAGGATTGATAATG
TAATAGGATCAATGAATATAAACATATAAAATGATGATAATAATATTTATAGAATTGTGTAGAATTGCAGATT
CCCTTTTATGGATTCCTAAATCCTTGAGGAGAACTTCTAGTATATTCTGTATACCTAATATTATAGCCTTTAT
CAACAATGGAATCCCAACAATTATCTCAACATTCACCCATTTCTCA
