# Supplementary figures and images for: Quadruplex-Forming Motif Inserted into 3′UTR of Ty1his3-AI Retrotransposon Inhibits Retrotransposition in Yeast
Source: Biology (Basel). 2021 Apr 20;10(4):347. doi: 10.3390/biology10040347 (PMC8074290; doi:10.3390/biology10040347)

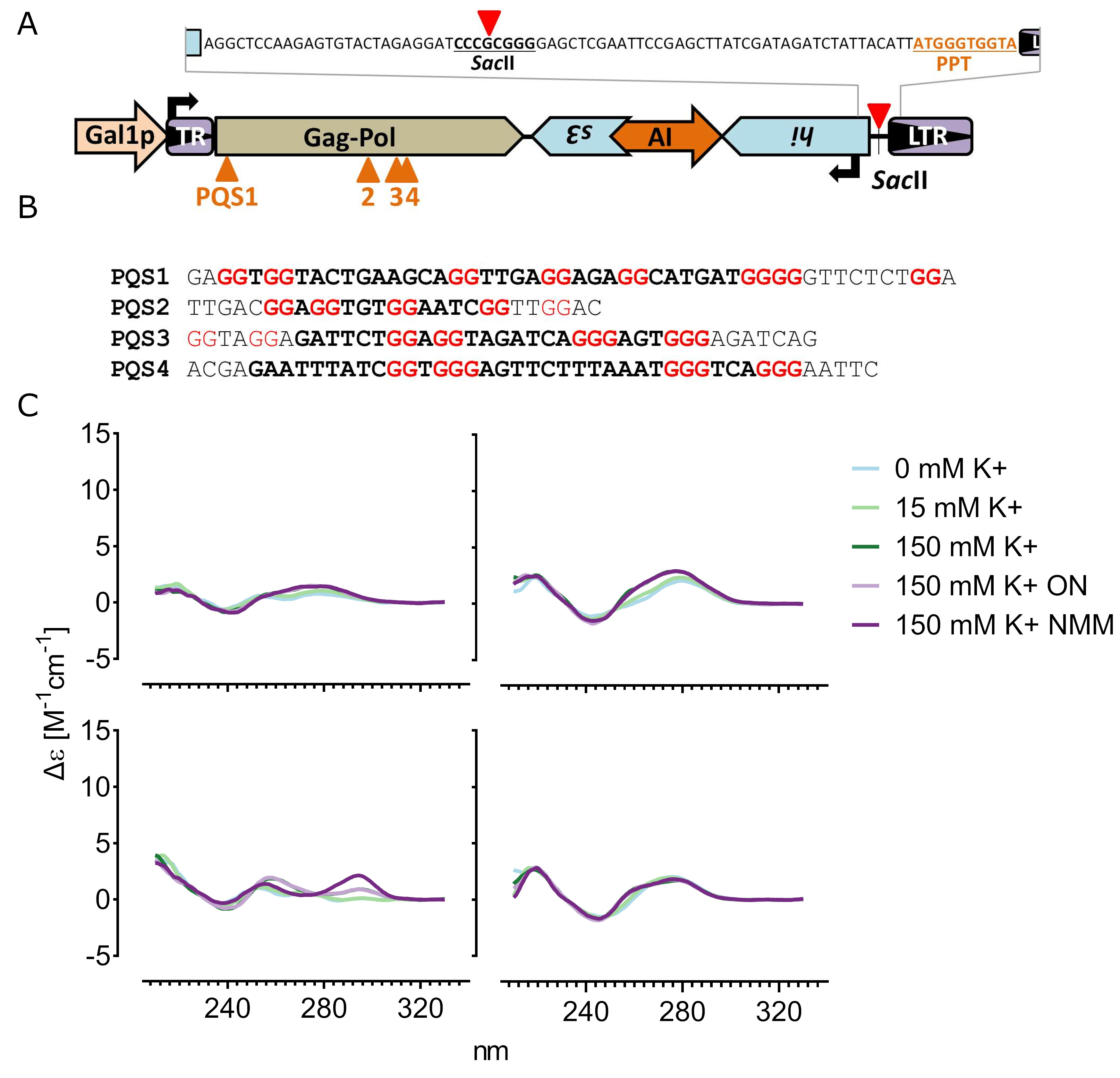

Supplement: Supplementary file 1 [file biology-10-00347-s001.zip › Fig S1 600dpi v2.jpg]

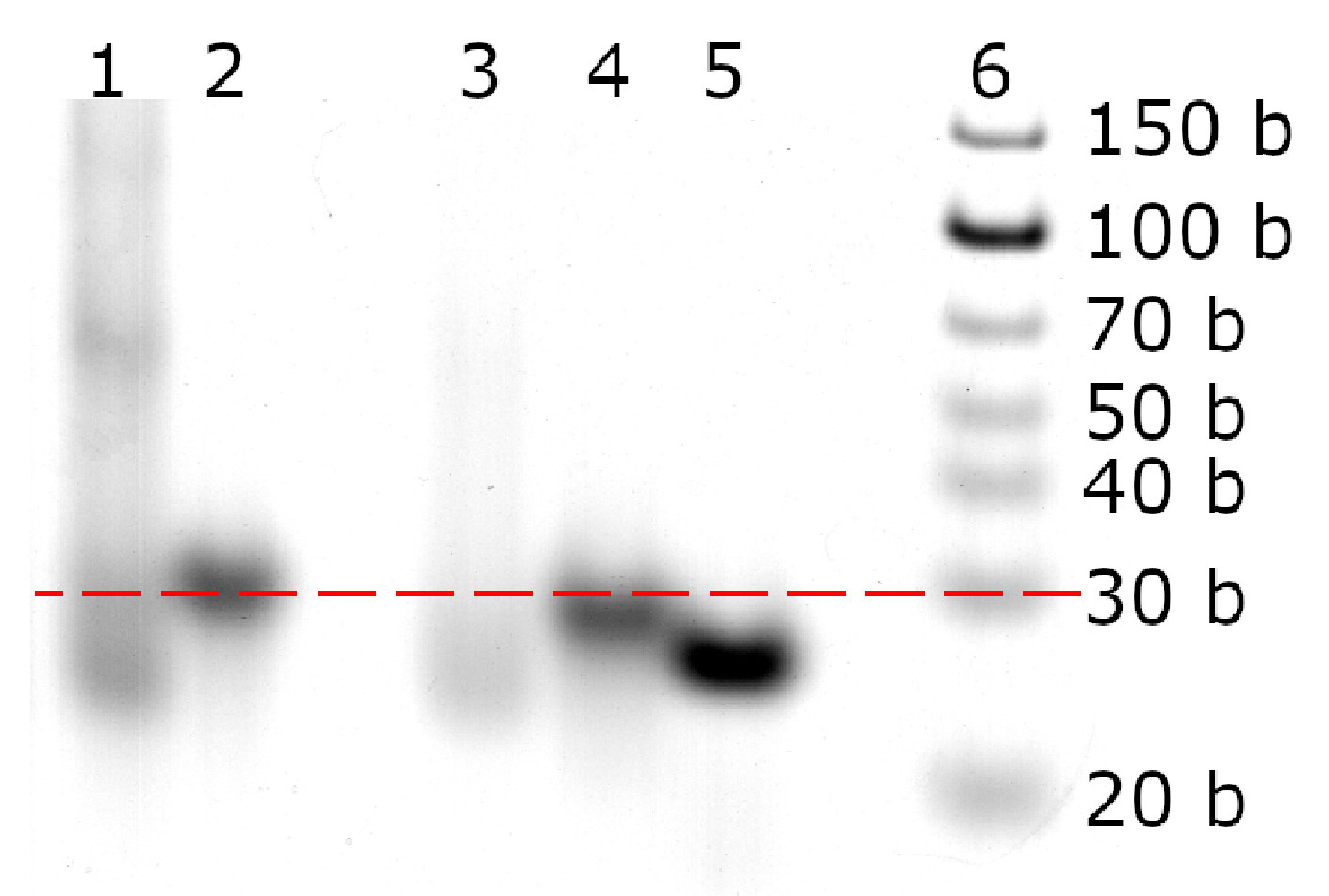

Supplement: Supplementary file 1 [file biology-10-00347-s001.zip › Fig S2 600dpi v1.jpg]

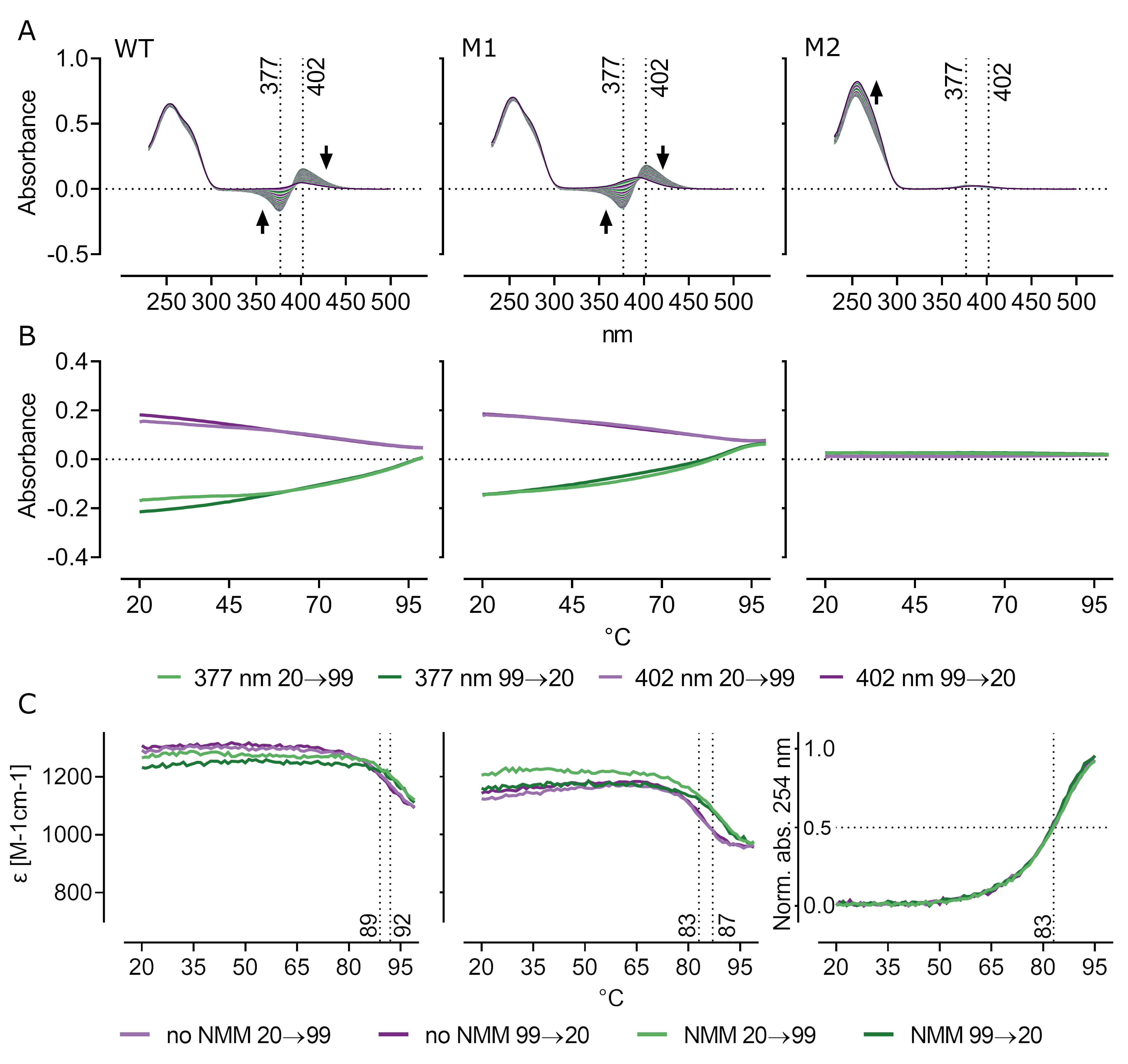

Supplement: Supplementary file 1 [file biology-10-00347-s001.zip › Fig S4 600dpi V2.jpg]

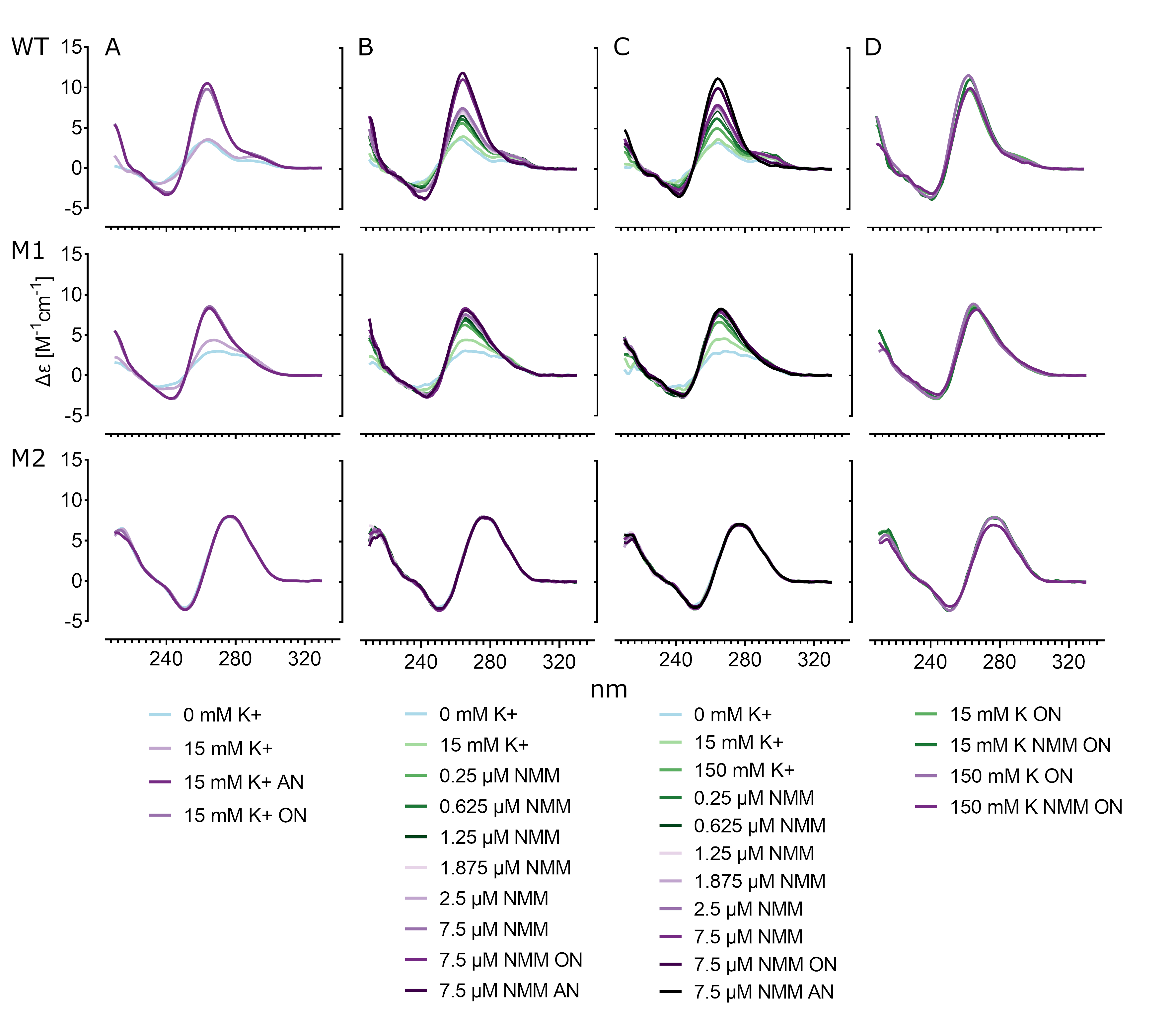

Supplement: Supplementary file 1 [file biology-10-00347-s001.zip › FigS3 600dpi v3.jpg]
